# Photosensitizer-based multimodal PSMA-targeting ligands for intraoperative detection of prostate cancer

**DOI:** 10.7150/thno.52166

**Published:** 2021-01-01

**Authors:** Yvonne H.W. Derks, Mark Rijpkema, Helene I.V. Amatdjais-Groenen, Annemarie Kip, Gerben M. Franssen, J. P. Michiel Sedelaar, Diederik M. Somford, Michiel Simons, Peter Laverman, Martin Gotthardt, Dennis W. P. M. Löwik, Susanne Lütje, Sandra Heskamp

**Affiliations:** 1 Department of Medical Imaging, Nuclear Medicine, Radboud University Medical Center, Radboud Institute for Molecular Life Sciences, Nijmegen, The Netherlands.; 2 Radboud University Nijmegen, Institute for Molecules and Materials, Systems Chemistry, Nijmegen, The Netherlands.; 3 Department of Urology, Radboud University Medical Center, Nijmegen, The Netherlands.; 4 Prosper Clinics, Nijmegen, The Netherlands.; 5 Department of Urology, Canisius Wilhelmina Hospital, Nijmegen, The Netherlands.; 6 Department of Pathology, Radboud University Medical Center, Nijmegen, The Netherlands.; 7 Department of Nuclear Medicine, University Hospital Bonn, Bonn, Germany.

**Keywords:** Prostate-specific membrane antigen, imaging agents, prostate cancer, intraoperative, radiochemistry

## Abstract

Incomplete resection of prostate cancer (PCa) occurs in 15%-50% of PCa patients. Disease recurrence negatively impacts oncological outcome. The use of radio-, fluorescent-, or photosensitizer-labeled ligands to target the prostate-specific membrane antigen (PSMA) has become a well-established method for the detection and treatment of PCa.

**Methods:** Here, we developed and characterized multimodal [^111^In]In-DOTA(GA)-IRDye700DX-PSMA ligands, varying in their molecular composition, for use in intraoperative radiodetection, fluorescence imaging and targeted photodynamic therapy of PCa lesions. PSMA-specificity of these ligands was determined in xenograft tumor models and on fresh human PCa biopsies.

**Results:** Ligand structure optimization showed that addition of the photosensitizer (IRDye700DX) and additional negative charges significantly increased ligand uptake in PSMA-expressing tumors. Moreover, an *ex vivo* incubation study on human tumor biopsies confirmed the PSMA-specificity of these ligands on human samples, bridging the gap to the clinical situation.

**Conclusion:** We developed a novel PSMA-targeting ligand, optimized for multimodal image-guided PCa surgery combined with targeted photodynamic therapy.

## Introduction

Prostate cancer (PCa) ranks as the second most common cancer in men worldwide, affecting one in six males 1. Despite improvements in imaging methods, leading to better diagnoses, staging and therapy, PCa still causes substantial morbidity and mortality and is a leading cause of cancer-related deaths 2, 3. For patients with early stage disease, PCa treatment consists of active surveillance. In case of high-grade, locally advanced, or progressive disease, radical prostatectomy often is the curative treatment of choice, which may include extended lymph node dissection for patients with intermediate- or high-risk localized PCa 4. Unfortunately, surgical treatment of PCa faces two main challenges. First, close proximity of tumor lesions to vital anatomical structures, such as the bladder or neurovascular bundles, can impede wide local excision. This leads to positive surgical margins in 15% to 50% of patients, depending on the stage of the disease 5, 6. Second, translation of preoperative images to the surgical field remains difficult because of intraoperative tissue shifts, atypical location, and ambiguous morphology that hamper the detection of smaller tumor lesions 7, 8. Therefore, the ability to find and resect metastatic tumor tissue is currently based mainly on cognitive anatomical orientation and detection with the naked eye 8. Consequently, biochemical recurrences occur in up to 35% of these patients 9. These surgical challenges emphasize the significance of improved intraoperative metastatic lymph node detection, visualization of tumor margins and adjuvant ablative therapies. A promising strategy to overcome these obstacles is image-guided surgery combined with targeted photodynamic therapy (tPDT). tPDT is a method to induce cell death through administration and activation of a photosensitizer, causing the formation of reactive oxygen species (ROS) and singlet oxygen (^1^O_2_), both being highly toxic and short-lived radicals 10, 11.

To improve surgical treatment of PCa, recent developments have focused on prostate-specific membrane antigen (PSMA)-targeting ligands 12, 13. PSMA represents an excellent target for imaging of PCa, as its expression is drastically elevated in 90%-100% of local PCa lesions, tumorous lymph nodes, and metastatic bone lesions 14-16. PSMA ligands usually consist of a PSMA-binding motif (glutamate-urea-lysine, KuE) which can be functionalized with different imaging moieties such as chelators for radionuclide labeling or near-infrared fluorescent (NIRF) dyes for optical/fluorescence imaging. Differences in linker composition (e.g. linker length, hydrophilicity, charge) and the properties of the added functional groups for imaging can have a significant impact on the pharmacokinetics and binding affinity of PSMA ligands 17-19.

To aid the surgeon in complete resection of all cancerous tissue, PSMA ligands suited for both fluorescent and radionuclide detection techniques hold great promise. Due to its high penetration depth, radioguidance is especially suitable for the detection and guidance towards metastatic lesions. Nevertheless, exact tumor delineation based on the radiosignal remains difficult. Therefore, the fluorescent label can be used to directly visualize the tumors during surgery, opening up the opportunity for resections without positive surgical margins. Hence, an intraoperative multimodal imaging approach integrates the sensitive detection and quantitative capacity of nuclear-based detection methods with the high spatial resolution and accurate delineation potential of fluorescence imaging 20-23. In addition, photodynamic agents have the potential of combining fluorescence imaging for image-guided surgery with ablation of cancer cells induced by tPDT. Labelling multimodal PSMA ligands with a photosensitizer enables intraoperative tumor detection, delineation and tPDT.

In this study, we developed ligands for a new surgical approach in the treatment of PCa consisting of three phases. First, surgical guidance toward distant tumor lesions and metastatic lymph nodes will be performed via radiodetection of indium-111 (^111^In) using a gamma probe. Second, IRDye700DX-mediated NIRF imaging will be performed to visualize and delineate the tumor, as well as to mark potential residual tumor areas for tPDT. Last, end of surgery tPDT will be applied for tumor-specific destruction of tumor remnants. These include positive surgical margins and other residual tumor lesions that were deemed unresectable, for example due to proximity to other vital structures including the rectum, sphincter, neovascular bundles and the bladder.

Attachment of a complex, relatively large photosensitizer molecule such as IRDye700DX to small PSMA ligands is chemically challenging and may alter the biodistribution of such molecules significantly. To investigate this, we synthesized and characterized 13 novel multimodal PSMA-targeting ligands with various linkers conjugated with a photosensitizer and a chelator for ^111^In radiolabeling. The newly developed multimodal ligands were characterized using PSMA-expressing tumor cells, xenograft models, and human PCa tumor biopsies.

## Materials and Methods

### Synthesis of multimodal ligands

Different variants of glutamate-urea-lysine-based PSMA-targeting ligands, varying in their linker moieties, were synthesized using solid phase chemistry, coupled to IRDye700DX, and labeled with ^111^In. A detailed description of the synthetic procedures and chemical analyses (HPLC, ESI-ion trap, MALDI-ToF) is provided in the supplemental information (Supplementary Materials and Methods and Results, Figure S1, Figure S2, Figure S8).All amino acids were purchased from Iris Biotech GmbH or Fluorochem unless stated otherwise. DOTA-NHS and DOTAGA anhydride were purchased from CheMatech SAS. IRDye700DX was purchased from LI-COR Biosciences. Other reagents were purchased from Fisher Scientific unless stated otherwise and were reagent grade. All water used was highly pure (18 mΩ) and all HPLC-grade solvents were purchased from Biosolve B.V. Identity of the peptides was confirmed using high-resolution mass spectra recorded on a JEOL AccuTOF (ESI-MS), and the dye-conjugated peptides with MALDI-ToF Mass spectrometry on a Bruker Microflex LRF MALDI-ToF.

### Cell culture

Cell lines were purchased from the American Type Culture Collection. LS174T colon carcinoma cells were stably transfected with human PSMA using the plasmid pcDNA3.1-hPSMA and cultured in RPMI 1640 medium supplemented with 2 mM glutamine, 10% FCS, and 0.3 mg/ml G418 at 37 °C in a humidified atmosphere with 5% CO_2_ 5.

### Radiolabeling

Peptides were radiolabeled with ^111^InCl_3_ (Curium) in 0.5 M 2-(N-morpholino)ethanesulfonic acid (MES) buffer (twice volume of ^111^InCl_3_), pH 5.5, for 10-30 min at 45 °C under metal-free conditions 24. Following incubation, 50 mM ethylenediaminetetraacetic acid (EDTA) was added to a final concentration of 5 mM to chelate unincorporated ^111^InCl_3_. Labeling efficiency was determined by instant thin-layer chromatography (ITLC) using silica gel-coated paper (Agilent Technologies) and 0.1 M ammonium acetate containing 0.1 M EDTA, pH 5.5, as the mobile phase. Moreover, radiochemical purity was checked using reverse-phase high performance liquid chromatography (RP-HPLC) on an Agilent 1200 system (Agilent Technologies) with an in-line radiodetector. A C18 column (5 µm, 4.6 × 250 mm; HiChrom) was used at a flow rate of 1 ml/min with the following buffer system: buffer A, triethylammonium acetate (10 mM, pH 7); buffer B, 100% methanol; and a gradient of 97% to 0% buffer A (35 min). Peptides were purified by a Sep-Pak C18 light cartridge (Waters) and eluted from the cartridge with 50% ethanol in water.

### *In vitro* assays

Binding and internalization characteristics of all ligands were compared using LS174T-PSMA and wildtype LS174T cells. Cells were cultured to confluency in 6-wells plates followed by incubation at 37 °C for 2 h in 1 ml binding buffer (RPMI/0.5% BSA) with 50,000 counts per minute (cpm) of ^111^In-labeled ligand (0.1-0.25 pmol/well). Nonspecific binding was determined by coincubation with 2-(phosphonomethyl)pentane-1,5-dioic acid (2-PMPA, 21.57 µM). PSMA-specific binding was defined as nonspecific binding subtracted from total binding. To retrieve the membrane-bound fraction, cells were washed with PBS twice and incubated with acid buffer (0.1 M acetic acid, 154 mM NaCl, pH 2.6) for 10 minutes at 0 °C. After incubation, the membrane bound fraction (in acid buffer) was collected, cells were washed, lysed with 0.1 M NaOH and cell lysis (intercellular activity) was collected. Membrane-bound activity and intercellular activity fractions were measured in a gamma-counter (2480 WIZARD^2^ Automatic Gamma Counter, PerkinElmer) 5, 25. Human serum was used to determine the *in vitro* serum stability of the multimodal ligands (0.8 µg/ml serum, 5 MBq/μg labeling). After incubation for 2 h at 37°C, serum proteins were precipitated by adding a 1:1 (v/v) volume of acetonitrile followed by 5 min of centrifugation at 3220×g. Stability was evaluated by RP-HPLC analysis after 2 h of incubation at 37 °C. Equilibrium binding constant (K_d_) and 50% inhibitory concentration (IC_50_) values of the ligands were determined in competitive binding assays. Experimental details are described in the supplementary materials and methods.

### Lipophilicity

Lipophilicity (logD values) of all radiolabeled ligands was determined by adding 300,000 cpm (0.6-1.5 pmol) to a mixture of 3 ml PBS and 3 ml n-octanol. Tubes were vortexed vigorously for 1 min and centrifuged for 5 min at 201×g for phase separation. The concentration of radioactivity in a defined volume of each layer was measured in a well-type gamma-counter.

### Animal tumor model

Animal experiments were performed in 8-10 weeks old male BALB/c nude mice (Janvier). Animals were housed in individually ventilated cages (Blue line IVC, 4-5 mice per cage) under nonsterile standard conditions with cage enrichment present and free access to chlorophyll-free animal chow (Sniff GmbH) and water. Mice were subcutaneously inoculated with 3.0 x 10^6^ LS174T-PSMA cells in the left flank and 1.5 × 10^6^ LS174T cells in the right flank, diluted in 200 μL of complete RPMI 1640 medium. Eleven days after tumor cell inoculation when xenografts were approximately 0.1 cm^3^, tracers were injected intravenously via the tail vein. The researchers were not blinded for the experimental groups and tumor-bearing mice were block-randomized into groups based on tumor size. All experiments were approved by the institutional Animal Welfare Committee of the Radboud university medical center and were conducted in accordance to the guidelines of the Revised Dutch Act on Animal Experimentation.

### Dose and time optimization

Four groups of five mice received an intravenous injection of 1 MBq ^111^In-PSMA-N064 diluted in PBS/0.5% BSA. The effect of the ^111^In-PSMA-N064 dose on the biodistribution of ^111^In-PSMA-N064 was studied by administering various peptide doses: 0.1, 0.3, 1 or 3 nmol/mouse. Two hours post injection, mice were euthanized using CO_2_/O_2_-asphyxiation and the biodistribution of ^111^In-PSMA-N064 was determined by counting relevant tissues after dissection. Five additional mice were co-injected intravenously with a 100-fold molar excess of unlabeled PSMA-617 (10 nmol/mouse) to determine the non-specific uptake of ^111^In-PSMA-N064.To find the optimal imaging timepoint for ^111^In-PSMA-N064, four groups of five mice received an intravenous injection of 10 MBq ^111^In-PSMA-N064 (molar activity 33.3 MBq/nmol) in PBS/0.5% BSA. At 1, 2, 4, and 24 h post injection, mice underwent µSPECT/CT and NIRF imaging followed by dissection. Tumors, blood, and relevant organs and tissues were dissected, weighed, and radioactivity in each sample was quantified using a well-type gamma-counter. The results were expressed as percentage of injected dose per gram of tissue (%ID/g).

### Dual-modality µSPECT/CT, NIRF imaging and biodistribution

Mice were injected intravenously with 10 MBq ^111^In-labeled PSMA ligands (0.3 nmol, molar activity 33.3 MBq/nmol) in PBS/0.5% BSA. Two hours post injection, mice were euthanized by CO_2_/O_2_-asphyxation and images were acquired with the IVIS fluorescence imaging system (Xenogen VivoVision IVIS Lumina II, Caliper Life Sciences), using an acquisition time of 10 s. Subsequently, µSPECT/CT images were acquired (U-SPECT II, MILabs) with a 1.0 mm diameter pinhole mouse collimator tube 26. Mice were scanned for 30 min followed by a CT scan (spatial resolution 160 μm, 65 kV, 615 μA) for anatomical reference. µSPECT/CT scans were reconstructed with MILabs reconstruction software, using an ordered-subset expectation maximization algorithm, energy windows 154 - 188 keV and 220-270 keV, 3 iterations, 16 subsets, voxel size of 0.4 mm. SPECT/CT scans were analyzed and maximum intensity projections (MIPs) were created using the Inveon Research Workplace software version 4.1 (Siemens Preclinical Solutions). NIRF images were analyzed using Living Image software version 4.2 (Caliper Life Sciences). Tissues of interest were dissected, weighed and measured for radioactivity in a gamma-counter as described above. In the fluorescence-guided surgery feasibility study, pre-operative imaging was performed to visualize tumor tissue. After white light resection, NIRF imaging was repeated to assess if any remaining tumor was present.

### *Ex vivo* incubation human biopsies prostatectomy

Ten patients who underwent robot-assisted radical prostatectomy with or without pelvic lymph node dissection were included. Fresh biopsies from the tumor and contralateral healthy region were taken directly after surgery. Biopsies were incubated at 37 °C, 5% CO_2_ for 4 h in 3 mL binding buffer (RPMI 1640 containing 0.1% w/v BSA) with 0.08 nmol of ^111^In-PSMA-N064 or ^111^In-PSMA-N140 (molar activity 26.3 MBq/nmol). Subsequently, biopsies were washed in 1 L PBS/0.1% BSA for 2 h followed by whole biopsy fluorescence imaging using a flatbed fluorescence scanner (Odyssey; channel, 700 nm; focus, 0.5 mm). Moreover, biopsies were exposed to a phosphor imaging plate for 12 h and a Typhoon FLA 7000 (GE Healthcare) phosphor imager was used for readout. Images were analyzed with Aida Image Analyzer, version 4.21 (Elysia-Raytest). Tumor regions within the tumor biopsy, based on the hematoxylin and eosin-stained cryosections of the biopsies, were drawn by a pathologist blinded for other outcomes of the study. The study was performed in accordance with the Code of Conduct of the Federation of Medical Scientific Societies in the Netherlands and with the 1964 Helsinki declaration and its later amendments or comparable ethical standards. The local institutional ethics committee of the Radboud university medical center approved this study (case number: 2018-5054). All samples and corresponding data were handled and stored anonymously.

### Fluorescence and radionuclide imaging of cryosections

Murine LS174T-PSMA tumors and biopsies taken after radical prostatectomy were snap frozen using Tissue-Tek (Sakura). Tumor cryosections (4 µm) were used for fluorescence imaging (Odyssey; channel, 700 nm; focus, 1.0 mm) and subsequently fixed with 4% paraformaldehyde followed by autoradiographic analysis after 10 days of exposure to a phosphor imaging plate (Typhoon FLA 7000, GE Healthcare).

### Immunohistochemistry

Murine LS174T-PSMA tumors and biopsies taken after radical prostatectomy were snap-frozen using Tissue-Tek (Sakura). Tumor cryosections (4 µm) were immunohistochemically stained for PSMA with anti-PSMA antibody (1:200 dilution, rabbit monoclonal, EPR6253, Abcam). Briefly, antigen retrieval was performed with 10 mM citrate pH 6.0 in an antigen retrieval module (PT-Module, 10 min, 96°C). Endogenous peroxidase activity was quenched with 0.03% H_2_O_2_ for 10 min and sections were preincubated with 20% (v/v) normal goat serum. Subsequently, sections were incubated with rabbit anti-PSMA antibody (1:200) overnight at 4 °C. Sections were washed with 10 mM PBS and incubated with biotinylated goat-anti-rabbit antibody (1:400, Vector) for 30 min at RT followed by incubation with Vectastain Elite ABC kit (Vector Laboratories) for 30 min. Bound antibodies were visualized using diaminobenzine (Sigma-Aldrich). All slides were counterstained with hematoxylin (Klinipath) for 5 seconds and mounted with a cover slip (permount, Fisher Scientifc). On adjacent sections, standard hematoxylin and eosin staining was performed.

### *In vitro* targeted photodynamic therapy

LS174T-WT and LS174T-PSMA cells were cultured to confluency in 48-well plates. Cells were incubated for 2 h (5% CO_2_, 37 °C) with 0 or 30 nM PSMA ligand in binding buffer (RPMI 1640 medium with 0.5% BSA) in triplicate. The triplicates were randomly distributed over the center of the plates considering the variation in light intensity within the NIR LED device 27. As negative control for NIR light irradiation effects, cells that only received PBS without PSMA ligand were included. After washing with PBS, 0.5 ml fresh binding buffer was added to each well. Subsequently, cells were irradiated with a NIR LED that emits light at a wavelength of 670 to 710 nm 27. The typical forward voltage was 2.6 V, creating a power output of 490 mW using 126 individual LED bulbs to ensure homogenous illumination of the area of interest predefined as 5 × 3 cm. The cells were irradiated at NIR radiant exposures of 100 J/cm^2^ and subsequently incubated for 1 h at 37 °C. As control for cellular toxicity of the PSMA ligands, cells incubated with PSMA ligand that were not irradiated with NIR light were also included as an experimental group. Cytotoxic effects of PDT with PSMA ligands were determined with a CellTiter-Glo^®^ assay (Promega Benelux) according to the manufacturer's instructions. Binding buffer was replaced with 100 µl fresh binding buffer and 100 µl CellTiter-Glo^®^ 2.0 Assay. Plates were shaken (2 min) and incubated for 10 min at room temperature. To determine the metabolic activity of the cells, the luminescence was measured in a plate reader (Infinite^®^ 200 PRO, Tecan).

### Statistical analysis

Statistical analyses were performed with Graphpad Prism, version 5.03. Results are presented as mean ± SD. Differences in *in vitro* affinity and *in vivo* tumor and organ uptake were tested for significance using a one-way ANOVA with a Bonferroni's multiple comparison post-test. Differences in fluorescence signal between the tumor regions and healthy regions of the *ex vivo* biopsies were also tested using a one-way ANOVA and Bonferroni's posttest. Differences were considered significant at *P* < 0.05, two-sided.

## Results

### Design of multimodal PSMA ligands

We designed glutamate-urea-lysine-based PSMA ligands with various linkers and conjugated them with DOTAGA or DOTA and IRDye700DX. The ligands are further referred to as [^111^In]In-DOTA(GA)-IRDye700DX-PSMA or Nxxx ligands. The most basic ligand, N025, consists of 6-aminohexanoic acid (AhX), d-phenylalanine (*d*-Phe) and a lysine modified on its N-terminus with DOTAGA as a metal chelator (Table 1). Next, we attached IRDye700DX to obtain N046. As it has been reported that negative charges improve the PSMA-binding and tumor-to-background ratios of related PSMA ligands 18, 19, we incorporated glutamic acid residues in the linker (N057b, N064, N142). Other adaptation of the linker part include the introduction of short PEG_4_ spacers (NJ26, NJ27 and N111), replacement of DOTAGA by DOTA (N122, N140) and removal of the *d*-Phe (N143, N144). As a control, we designed a ligand similar to N064 that is lacking the glutamic acid in the PSMA-binding motif (referred to as N064-Incomplete (N064inc)). All ligands described in this study are summarized in Table 1.

**Figure 1:**
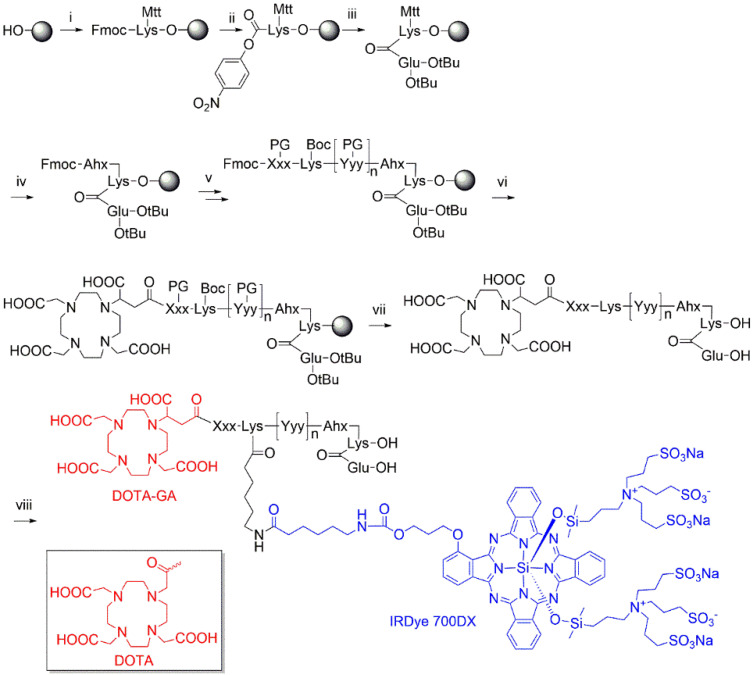
** Synthesis of DOTA(GA)-IRDye700DX-PSMA multimodal ligands.** i) Fmoc-Lys(Mtt)-OH, DIPCDI, DMAP; ii) p-nitrophenol chloroformate, DiPEA; iii) H-Glu(OtBu)-OtBu, DiPEA; iv) a) piperdine, b) Fmoc-Ahx-OH, DIPCDI, HOBt; v) multiple steps: a) piperdine, b) Fmoc-Xxx(PG)-OH, DIPCDI, HOBt; vi) DOTAGA anhydride or DOTA-NHS, DiPEA; vii) TFA; vii) IRDye700DX-OSu, DiPEA.

Multimodal ligands were synthesized on solid phase, except for the attachment of the IRDye700DX, as the dye was not stable under the acidic conditions required to cleave the ligands from the resin and to remove the protective groups (Figure 1). The synthesis started with a reaction of immobilized Mtt-protected lysine with p-nitrophenol chloroformate and DiPEA as a base, followed by substitution of the p-nitrophenol group by glutamic acid di-tert-butyl ester to obtain the PSMA-binding motif. After removal of the Mtt group, repeated couplings of the appropriate Fmoc-protected amino acid building blocks introduced the various linkers. At the N-terminus of the linker we attached either DOTA or DOTAGA as a metal chelator. The introduction of IRDye700DX on all compounds, except N025 and N057b, via its NHS ester completed the synthesis, after which the ligands were purified by reverse phase chromatography.

### Stability of multimodal PSMA ligands

Stability of IRDye700DX under ^111^In labeling conditions was measured at different time points using fluorescence measurements and HPLC. Labelling at 95 °C led to a severe loss in fluorescence signal, whereas after labeling at 45 °C the fluorescence signal was preserved, demonstrating instability of the IRDye700DX at high temperatures in combination with a pH of 5.5 required for the ^111^In labeling. Although the fluorescent signal remained stable at 45 °C, HPLC analysis demonstrated the presence of a second peak during the course of the labeling (10-30 min, pH 5.5), indicating that the ligands are partly unstable (Figure S3). Nonetheless, we collected both peaks separately and showed PSMA-affinity of the ligands that represent each peak (Figure S3).

### Linker modifications alter binding affinity and uptake of multimodal ligands *in vitro*

**Figure 2:**
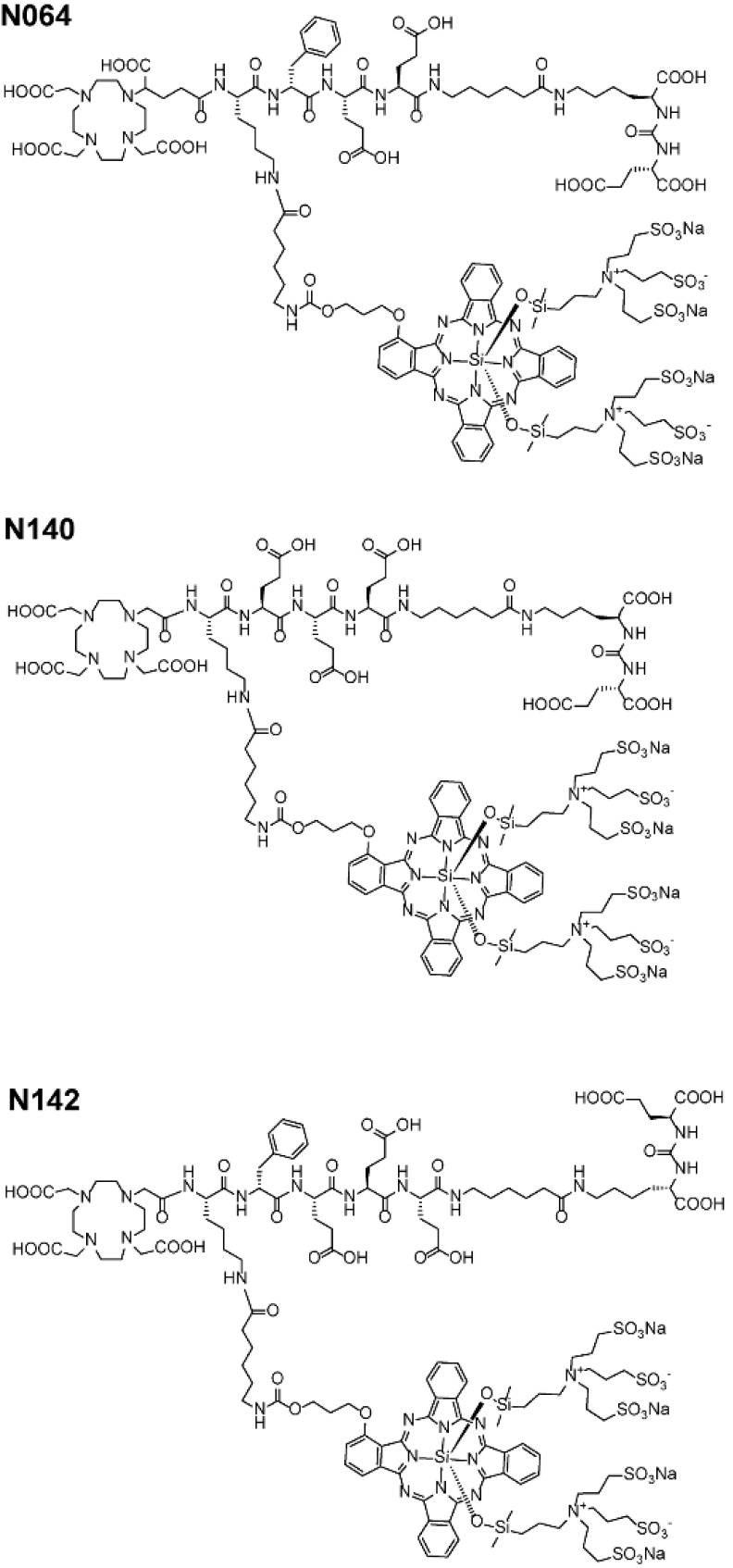
Structure of best performing N064, N140 and N142 multimodal ligands.

We first verified the PSMA-binding potential of our ligands in an *in vitro* binding and internalization assay using PSMA-expressing LS174T cells, in which all multimodal ligands showed specific binding (Figure S4). Direct comparison of the ligands revealed that N064 and N142 had the highest membrane-bound and internalized fraction (Table 1, Figure 2). As expected, we observed no binding and internalization upon incubation with control ligand N064inc, demonstrating the necessity of an intact PSMA-binding motif (KuE) for PSMA binding. Addition of neutral spacers (NJ26, NJ27, N111), replacement of DOTAGA by DOTA (N122, N140), and removal of the *d*-Phe (N143, N144) did not improve binding. We observed a remarkable increase in internalization of ligand N064 compared with the N057b ligand without the IRDye700DX, suggesting the involvement of IRDye700DX in the internalization of the multimodal PSMA ligands. The LogD values of the ligands ranged from -2.3 to -2.8, and were lowest in ligands without IRDye700DX. Furthermore, N064, N111 and N142 were stable in human serum (2 h, 37°C). We measured the IC_50_ and the K_d_ of N064, which did not differ significantly from the clinically available PSMA ligand [^111^In]In-DOTA-PSMA-617 (PSMA-617, Table 1).

### Multimodal ligands demonstrate PSMA-specific tumor uptake and rapid pharmacokinetics *in vivo*

**Figure 3:**
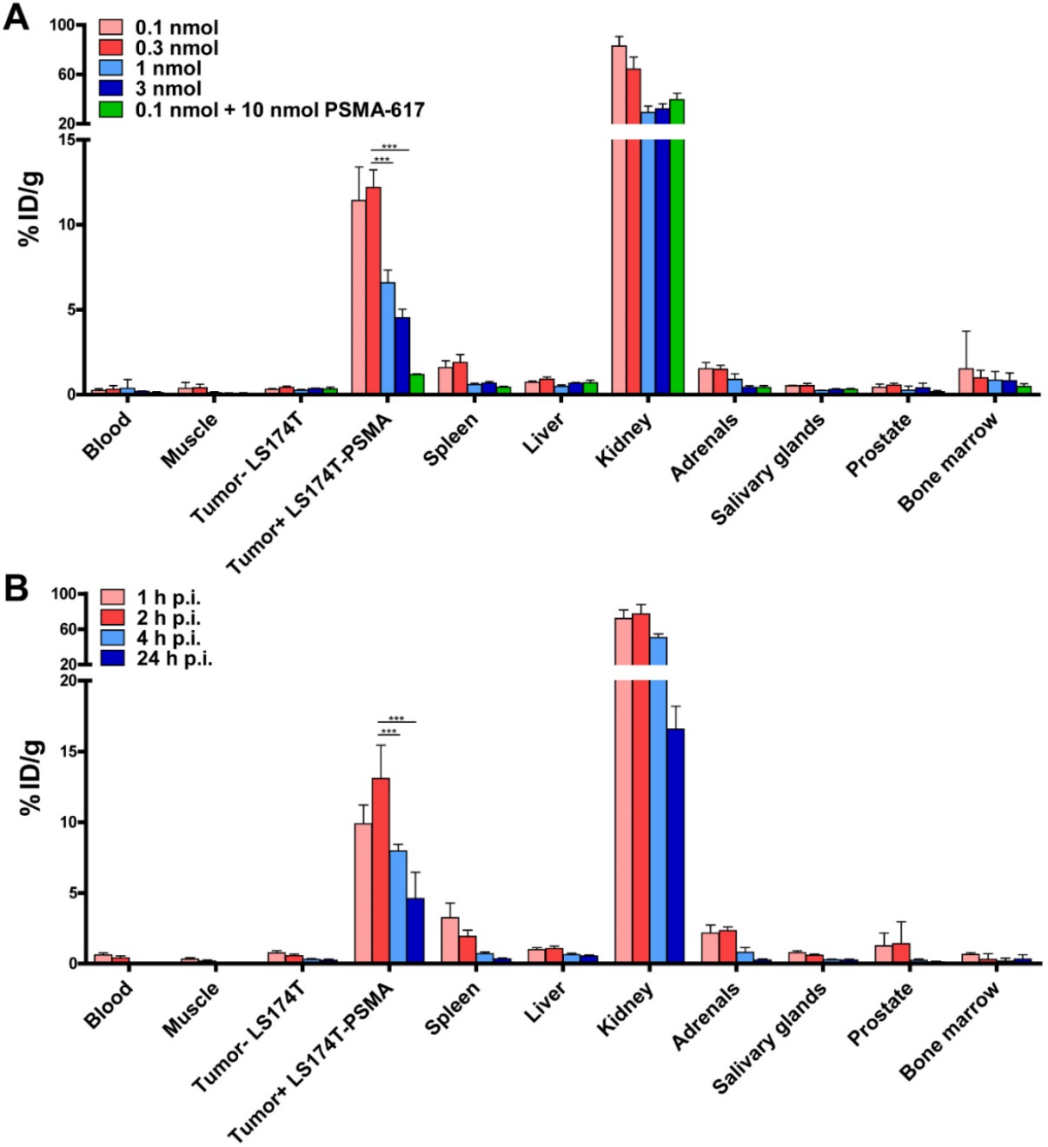
** Specific tumor uptake and rapid pharmacokinetics of N064 ligand. (A**) Dose optimization of multimodal ^111^In-labeled N064 ligand (0.1-3 nmol/mouse, 2 h p.i., 1 MBq/mouse, n=5/group) in mice bearing subcutaneous LS174T-PSMA and LS174T xenografts. As a control, 0.1 nmol of N064 was coinjected with a molar excess of unlabeled PSMA-617 (10 nmol). **(B)** Time optimization to determine the optimal timepoint for imaging and therapy (0.3 nmol/mouse, 10 MBq/mouse, n=5/group) in mice bearing subcutaneous LS174T-PSMA and LS174T xenografts. Biodistribution was determined after dissection at 1, 2, 4 and 24 h p.i, data is expressed as %ID/g ± SD. ^***^ Indicates *p* < 0.001.

Because of its promising *in vitro* characteristics, we performed dose and time optimization studies for ligand-mediated multimodal imaging of PSMA-expressing tumors using the N064 ligand. N064 demonstrated PSMA-specific accumulation in s.c. LS174T-PSMA tumors. The injected doses with highest specific tumor uptake were 0.1 and 0.3 nmol (Figure 3A), resulting in a tumor uptake of 11.4 ± 2.0 %ID/g and 12.2 ± 1.0 %ID/g, respectively. Tumor uptake in %ID/g was reduced significantly when the amount of tracer was increased to 1 and 3 nmol (*p*<0.001). However, absolute PSMA-positive tumor uptake continued to rise upon administration of higher dosages, going from 0.011 ± 0.002 nmol/g tumor (0.1 nmol injected dose) to 0.113 ± 0.012 nmol/g tumor (3 nmol injected dose, Figure S5). N064 also accumulated in the kidneys, the main excretory organ of these ligands (64.3 ± 9.7 %ID/g at 0.3 nmol dose). Co-injection of 0.1 nmol N064 with 10 nmol unlabeled PSMA-617 reduced tumor uptake from 11.4 ± 2.0 %ID/g to 1.2 ± 0.04 %ID/g (*p*<0.001), indicating PSMA-specificity of the tumor uptake. Moreover, co-injection of unlabeled PSMA-617 led to a 47% decrease in kidney accumulation, suggesting that uptake of the ligand in the kidneys was partially PSMA-specific. We observed low uptake of the ligand in all other organs, including the LS174T wildtype tumor, resulting in high tumor-to-organ ratios (Table S1). These results led us to select the 0.3 nmol dose to further investigate tracer pharmacokinetics, including the optimal timepoint for imaging (Figure 3B). Uptake in the tumor gradually increased up to 2 h post injection. Tumor uptake was 9.8 ± 1.3 %ID/g and 13.1 ± 2.3 %ID/g at 1 and 2 h after injection, respectively. Uptake significantly decreased from 2 to 4 h (8.0 ± 0.5 %ID/g, *p*<0.001)) and 24 h (4.6 ± 1.9 %ID/g, *p*<0.001).

### Addition of fluorophore and negative charges increase ligand uptake in PSMA-expressing tumors

**Figure 4:**
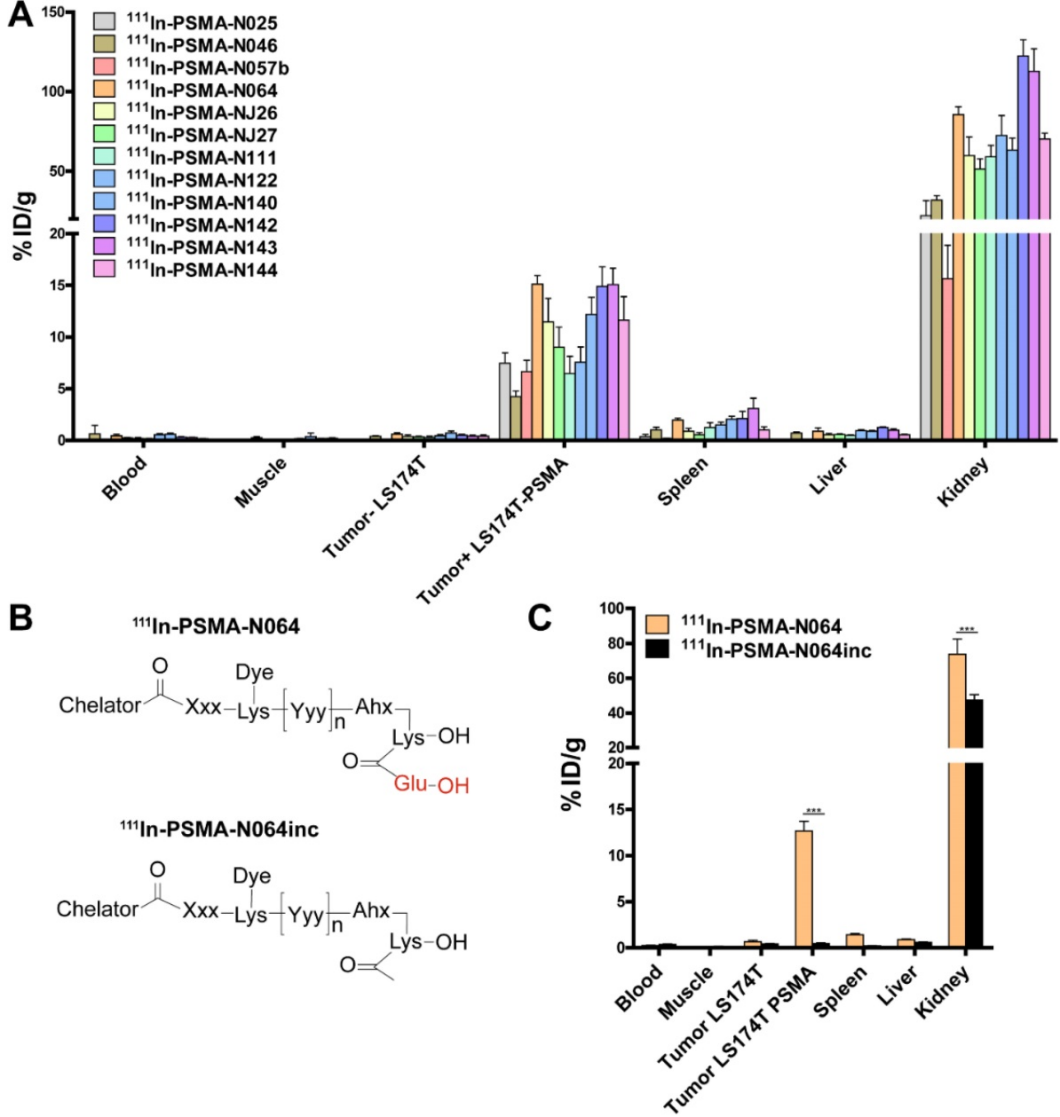
** Linker modifications in [^111^In]In-DOTA(GA)-IRDye700DX-PSMA multimodal ligands influence tumor uptake. (A)** Biodistribution of 12 multimodal ligands (0.3 nmol, 10 MBq/mouse, 2 h p.i., n= 5/group) in mice bearing subcutaneous LS174T-PSMA and LS174T wildtype xenografts as determined after dissection. **(B)** General structure of N064 and the control ligand N064inc, lacking the glutamic acid in the PSMA-binding motif.** (C)** Biodistribution of ^111^In-labeled N064 and control N064inc (0.3 nmol, 10 MBq/mouse, 2 h p.i., n= 5/group) in mice bearing subcutaneous LS174T-PSMA and LS174T wildtype xenografts. Data is expressed as %ID/g ± SD, ^***^ indicates *p* < 0.001.

To elucidate the importance of molecular composition on ligand accumulation in PSMA-expressing tumors, we compared tracer uptake of all ligands (0.3 nmol, 10 MBq/mouse, dissection at 2 h p.i.). All multimodal ligands specifically accumulated in s.c. PSMA-positive LS174T-PSMA tumors, with the highest uptake of 15.1 ± 0.8 %ID/g for the N064 ligand (Figure 4A). Uptake in PSMA-positive tumors was significantly higher compared with PSMA-negative tumors for all ligands (*p*<0.001). PSMA-positive tumor uptake was in line with the *in vitro* data (Figure S3). PSMA-positive tumor uptake of N064 (15.1 ± 0.8% ID/g) was significantly higher compared to a similar ligand without the fluorophore; N057b (6.7 ± 1.1% ID/g, *p*<0.001). Overall, addition of negative charges in the linker part of the ligand, use of DOTAGA as a chelator, and conjugation of the IRDye700DX moiety, enhanced uptake in the PSMA-positive tumors. We measured minimal uptake in the LS174T wildtype tumor, blood, muscle, liver, and spleen, leading to high tumor-to-organ ratios for all ligands (Table S2). Ligand uptake in the excretory organ, the kidneys, ranged between 15.6 ± 3.2 and 122.4 ± 10.2% ID/g for N057b and N142, respectively. From the three ligands with the highest tumor uptake, kidney accumulation of N064 was significantly lower compared with N142 and N143 (*p*<0.001).

Next, we synthesized N064inc as a control, a ligand similar to N064 lacking the glutamic acid in the PSMA binding motif (Figure 4B). In a separate experiment, we compared the biodistribution of this control ligand N064inc to that of N064 (Figure 4C). Tumor uptake of the control ligand (0.46 ± 0.2% ID/g) was significantly lower (1/27^th^) compared with the N064 ligand (*p*<0.001), demonstrating the necessity of an intact PSMA-binding motif for PSMA binding. Moreover, kidney accumulation of N064inc was significantly decreased as compared to N064 (*p*<0.001).

### Multimodal ligand-mediated µSPECT/CT and fluorescence imaging clearly visualize PSMA-positive tumors

**Figure 5:**
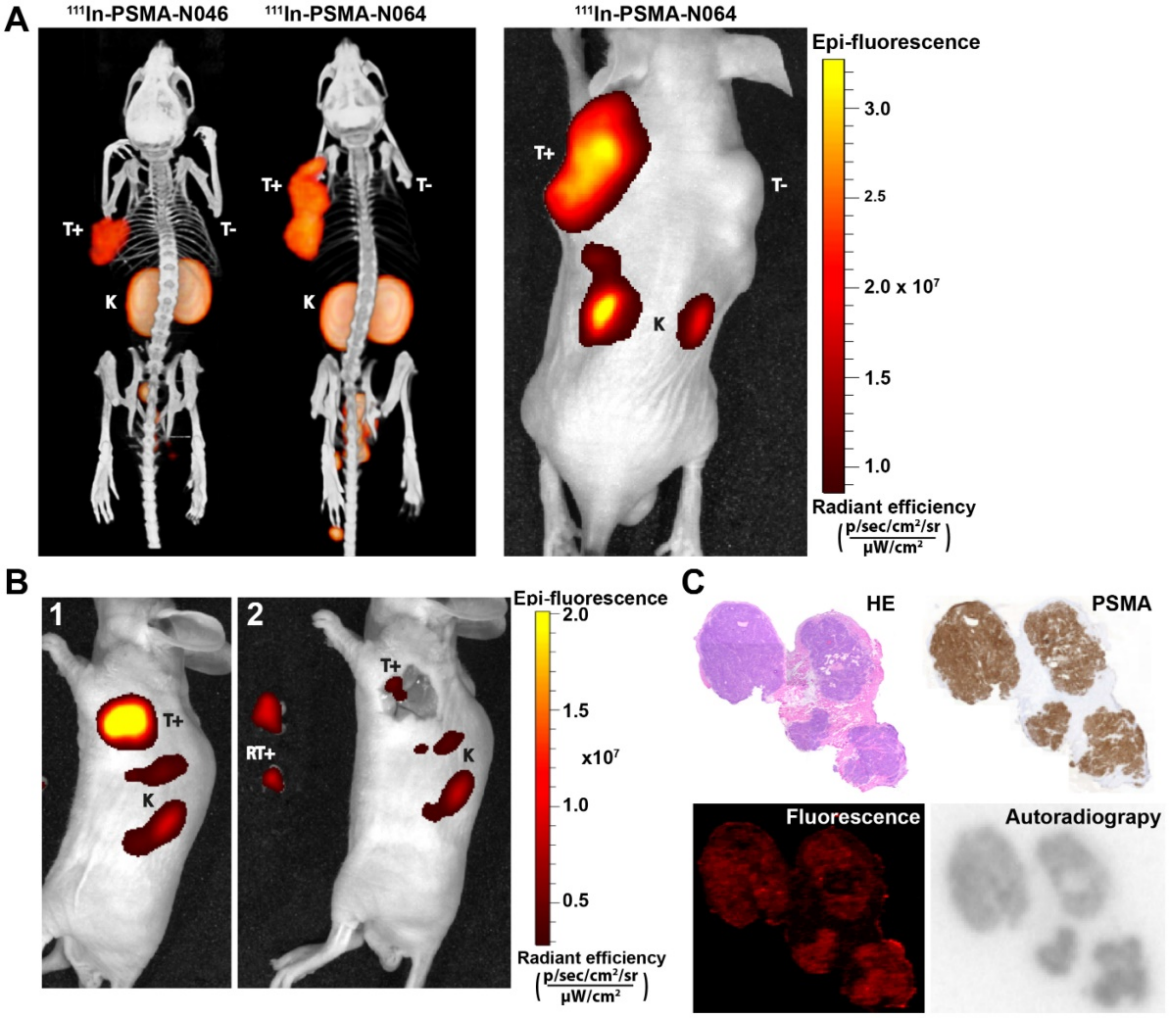
** Multimodal ligands clearly visualize PSMA-positive tumors using both radionuclide and fluorescence imaging, and may be used for image-guided resection of prostate cancer. (A)** µSPECT/CT (left) and NIRF (right) images of mice with s.c. LS174T-PSMA (T+) and wildtype LS174T (T-) tumors after i.v. injection of ^111^In-labeled N064 or N046 (0.3 nmol, 10 MBq/mouse, 2 h p.i.). Ligands are excreted via the kidneys (K). **(B)** NIRF-guided resection of a s.c. LS174T-PSMA tumor using N064: 1 NIRF image before resection, 2 NIRF imaging after resection revealed residual tumor tissue. RT+; Resected PSMA-positive tumor. **(C)** Colocalization of N064 fluorescence imaging, autoradiography and immunohistochemistry (PSMA and hematoxylin and eosin) in 4-µm tumor cryosections of s.c. PSMA-LS174T tumors (0.3 nmol/mouse, 2 h p.i.).

To determine the intraoperative imaging potential of our multimodal ligands, we scanned mice with a µSPECT/CT and near infrared fluorescence (NIRF) scanner. The subcutaneously growing LS174T PSMA-positive tumors were clearly visualized with all multimodal ligands using both imaging modalities. PSMA-negative LS174T tumors demonstrated lower ligand uptake. µSPECT/CT images of N046 (lowest tumor uptake) and N064 (highest tumor uptake), and the corresponding NIRF fluorescence image of N064, are shown in Figure 5A. µSPECT/CT images visualized differences in tumor uptake among all ligands and indicated high renal tracer accumulation in all mice (Figure S6). Moreover, the feasibility of PSMA-N064-mediated NIRF imaging to aid in achieving radical surgical resection is shown in Figure 5B, where NIRF imaging after white light resection of the tumor revealed small residual tumor tissue.

Cryosections of an s.c. PSMA-positive tumor are depicted in Figure 5C. Immunohistochemical analysis revealed PSMA expression within the tumor. An accurate microscopic colocalization of the radioactive ^111^In signal and the IRDye700DX fluorescent signal with this PSMA expression was observed.

### Multimodal ligands specifically accumulate in human prostate cancer biopsies

**Figure 6:**
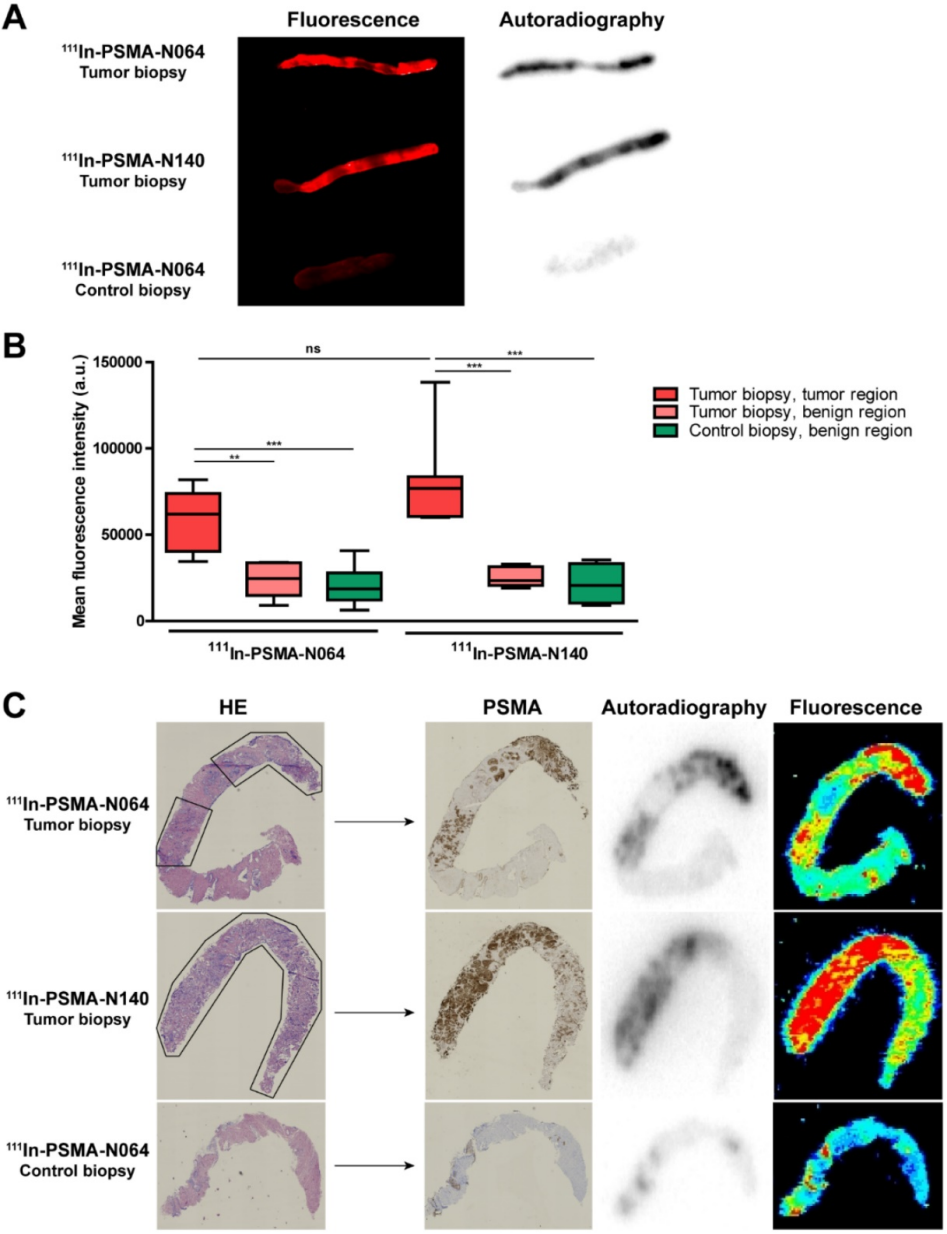
** High PSMA-specific uptake of ^111^In-labeled N064 and N140 ligands in human prostate biopsies taken after radical prostatectomy. (A)** Representative macroscopic fluorescence image and autoradiography of N064 incubated whole tumor biopsy, N140 incubated whole tumor biopsy and N064 incubated contralateral whole control biopsy. **(B)** Quantification of N064 and N140 incubated biopsies based on fluorescence images. Mean fluorescence intensity of tumor regions within the tumor biopsy were compared to fluorescence intensity in normal regions within the tumor biopsy and normal regions in the control biopsy, as defined by a pathologist. ^**^ *p* < 0.01, ^***^ *p* < 0.001, ns = not significant. **(C)** Colocalization of immunohistochemical staining (hematoxylin & eosin and PSMA) with autoradiography and fluorescence imaging within tumor regions (outlined in black) of 4-µm cryosections.

To examine the PSMA-specific accumulation of our ligands in human prostate cancer, tissue samples were incubated with ^111^In-labeled N064 and N140, the best performing DOTAGA and DOTA compounds, respectively (Figure 2, Figure 6). Patient characteristics are summarized in Table S3. Biopsies were taken from the tumor and healthy part of the prostate. Histopathological analysis confirmed the presence of tumor regions in the biopsies for 8 out of 10 patients. In two patients, no malignant tissue was detected in the tumor biopsies and they were excluded from the analysis. Macroscopic fluorescence imaging of the complete biopsies showed preferential accumulation of N064 and N140 in tumor tissue. Biopsies from normal prostate tissue incubated with N064 demonstrated lower fluorescent signal compared with tumor biopsies incubated with N064 (Figure 6A). As a result, a clear distinction between tumor and normal tissue was visible, and these results were consistent in all patients (Figure S7). Next to fluorescence imaging, the accumulation of these ligands in the tumor tissue was clearly demonstrated by autoradiography (Figure 6A). Visual assessment revealed colocalization of the fluorescent and radioactive signal in both tumor and normal prostate biopsies. Subsequently, ligand accumulation was quantified based on the fluorescent signal and did not significantly differ between the two ligands (*p* = 0.14, Figure 6B). A significant difference in mean fluorescence intensity was observed when comparing tumor regions of N064-incubated biopsies (mean fluorescence intensity; 58143 ± 17267) to either a normal tissue region within the same tumor biopsy (23712 ± 10364, *p*<0.01) or the contralateral control biopsy sample (20220 ± 10871, *p*<0.001). These significant differences were also observed in the N140-incubated biopsies when comparing tumor regions (mean fluorescence intensity; 80392 ± 27644) to either a normal tissue region within the same tumor biopsy and the contralateral control biopsy sample (25442 ± 5758 and 21317 ± 12003, respectively, *p*<0.001). Next, two different signal-to-noise ratios were calculated for each patient (Table S3). We calculated the ratio between fluorescent signal in the tumor and fluorescent signal in either a normal tissue region within the same tumor biopsy or the contralateral healthy prostate biopsy. The average ratio between signal in the tumor and signal in adjacent benign tissue was 2.7 ± 0.6 (range 2.1-3.6). The average ratio between tumor signal and the contralateral healthy prostate biopsy was 3.6 ± 1.7 (range 2.0-7.3).

Uptake of the multimodal ligands did not significantly differ between low grade (Gleason ≤ 3+4 = 7) and high grade tumors (Gleason ≥ 4+3 = 7, Figure S7). Cryosections depicted in Figure 6C demonstrate accurate microscopic colocalization of the marked tumor regions, outlined in black, with the radioactive signal, the fluorescent signal and PSMA expression.

### tPDT with multimodal ligands induces a PSMA-specific tumor cell destruction

**Figure 7:**
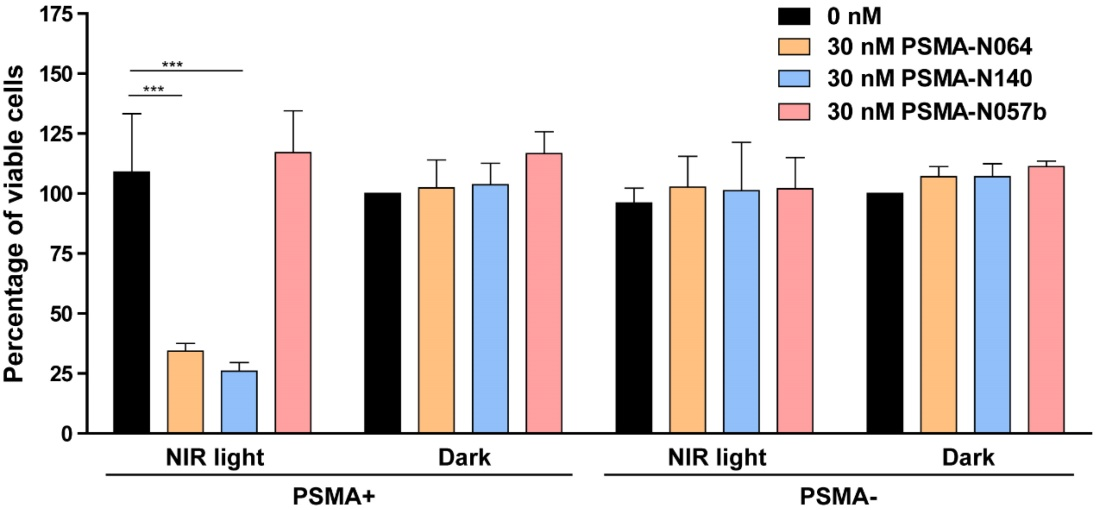
** tPDT efficacy of multimodal ligands *in vitro*.** Cell viability of LS174T-PSMA (PSMA+) and LS174T wildtype (PSMA-) cells following incubation with 0 or 30 nM of PSMA-N064, PSMA-N140 or PSMA-N057b, after either a 100 J/cm^2^ radiant exposure or no light exposure (dark). ^***^ Indicates *p*<0.001.

To elucidate the targeted photodynamic therapy (tPDT) potential of our top performing ligands, we performed a tPDT experiment on PSMA-positive and PSMA-negative cell cultures. We compared the tPDT effects between N064, N140 and a control ligand N057b (Figure 7). N057b is similar to N064 but does not contain the photosensitizer IRDye700DX and is therefore not able to produce tPDT-induced ROS and ^1^O_2_ 10, 11_._ After incubation of cells with N064 or N140 and exposure to NIR light, a cell viability of 34% ± 3.2% or 26% ± 3.6% was measured, respectively. After incubation with PSMA-N057b a cell viability of 117% ± 17.5% was observed, indicating the necessity of IRDye700DX-conjugated ligands for tPDT. All other controls, consisting of irradiated PSMA-negative LS174T-WT cells and non-irradiated LS174T-PSMA and LS174T-WT cells did not show a loss in cell viability (Figure 7).

## Discussion

Despite advancements in the surgical treatment of PCa, recurrences occur frequently 6. The effect of incomplete resection can be profound as it may lead to avoidable salvage therapies and potential poorer oncological and functional patient outcomes 4. To improve the result of PCa surgery, we have developed novel multimodal PSMA-targeting ligands that allow highly specific tumor localization and visualization in both preclinical models and human prostate tumor tissue.

The glutamate-urea-lysine-based PSMA ligand structure optimization performed in our study showed that addition of the fluorophore/photosensitizer (IRDye700DX) and additional negative charges in the linker or chelator part of the ligand, significantly improved tumor targeting. Addition of IRDye700DX showed advantageous effects on internalization and tumor uptake. *In vivo* PSMA-positive tumor uptake of N064 was significantly higher than that of its similar ligand without the dye. There are several potential explanations for the improved targeting properties of the photosensitizer-conjugated ligands. Firstly, attachment of the dye more than doubled the molecular weight of the PSMA ligands, leading to a longer circulatory half-life as indicated by higher blood levels 2 h p.i. (Figure 4A, Table S2). Because of this slower clearance, the photosensitizer-conjugated ligands had more time to accumulate in the tumor. Secondly, *in vitro* experiments demonstrated a remarkable increase in internalization and accumulation when IRDye700DX was conjugated to the ligand compared with unconjugated ligands. Previous research has shown that an increased internalization of PSMA ligands can lead to improved tumor retention 28. Therefore, the tumor retention of the photosensitizer-conjugated ligands could have increased compared to the ligands without the dye. Finally, changes in charge and lipophilicity of the PSMA ligands upon addition of the dye might alter the affinity in a significant manner. Previously, addition of other fluorophores including IRDye78, IRDye800CW and DyLight800 to small molecule PSMA-ligands similarly increased tracer uptake 29, 30. These findings are consistent with the increasing evidence suggesting that *in vivo* tumor targeting is not only determined by the PSMA-binding motif in these molecules, but is also facilitated by properties such as charge, hydrophobicity, and overall molecular structure of the ligands 18, 19, 31.

In addition to the effects of the photosensitizer, we explored how the linker design of our small molecule PSMA ligands alters tracer performance* in vitro* and *in vivo*. Introduction of multiple additional negative charges induced a positive effect on the PSMA-binding properties of the ligands (N064, N142, N143). These findings are in line with previous research where introduction of multiple negative charges increased tumor uptake and reduced the non-specific background, thereby improving tumor-to-background ratios 18, 19. In contrast to previous literature describing the positive effects of linker elongation on PSMA affinity 18, 32, increasing the linker length between the two imaging moieties and the PSMA-binding motif did not lead to favorable effects in our study (NJ26, NJ27, N111).

K_d_ values described in literature for clinically used PSMA-11, PSMA-617 and PSMA-I&T range from 5-12 nM 33-37. The K_d_ value of N064 (6.9 nM) is in the same nanomolar range, indicating that the affinity of N064 is comparable to the clinically used PSMA ligands. Moreover, tumor uptake in PSMA-positive LNCaP xenografts described in literature for PSMA-11, PSMA-617 and PSMA-I&T range from 5-11% ID/g at 1 hr p.i., indicating a similar performance of N064 to preexisting ligands. Nonetheless, no direct comparison could be made due to the use of the LS174T-PSMA xenografts in this study compared with LNCaP xenografts used in literature 33-37. However, a direct comparison of the LNCaP and LS174T-PSMA xenograft models did not show major differences in PSMA-I&T tracer uptake between these models 38, strongly indicating that performance of ligand N064 was in a similar range to those of the clinically available ligands 33-37. However, before clinical application of the multimodal ligands described here, a good manufacturing practice (GMP)-grade ligand needs to be produced and toxicity testing should be performed.

We demonstrated that our best ligands - N064, N140 and N142 - were able to visualize PMSA-positive tumors using both radionuclide and fluorescence imaging in a pre-clinical model. Importantly, we measured very low uptake in PSMA-positive tumors when using our non-PSMA binding control ligand N064inc, demonstrating the need for an intact PSMA-binding motif that allows the PSMA-specificity of our ligands. Overall, the ligands with high tumor uptake also demonstrated higher uptake in normal organs such as spleen and kidney. However, for intraoperative imaging and tPDT, high absolute tumor accumulation of the ligands is essential for the sensitivity of fluorescence-guided surgery and the efficacy of tPDT, whereas uptake in other organs outside the surgical field is of less importance. PSMA-targeted tracers with a photosensitizer are designed to accumulate in PCa lesions and the fluorescence camera or light (normal or laparoscopic 680 nm laser) can be focused to the tumor site as well, meaning that PSMA-targeted intraoperative imaging and tPDT is highly precise 11, 39, 40.

In the present study we describe PSMA-specific accumulation of our photosensitizer-based tracers on fresh human tissue samples. Tumor regions within the biopsies were clearly distinguishable from normal prostate tissue both by fluorescence imaging and autoradiography, and on a macroscopic and microscopic level. Within the biopsies, a precise colocalization was observed between tumor regions based on histological assessment and the fluorescent signal, the radioactive signal, and PSMA expression. These results obtained in patient-derived samples bridge the gap between preclinical research and clinical applications of multimodal PSMA-ligands. Importantly, we used the IRDye700DX as a fluorophore in this study. This dye is also a photosensitizer, which means that these ligands can be used for multimodal image-guided surgery in combination with PSMA-targeted photodynamic therapy (PSMA-tPDT). This study describes the *in vitro* proof-of-concept for ligand-mediated PSMA-tPDT. However, to truly examine efficacy and specificity of PSMA-tPDT using our best ligand, future studies need to be performed. For this, an *in vitro*,* in vivo* and* ex vivo* setting will be used.

Multiple multimodal PSMA ligands have been described in literature 41-43. However, these ligands mostly consisted of fluorescent dyes such as the IRDye800CW and Cy5, which cannot be used for tPDT. More recently, the tPDT potential of photosensitizer-based PSMA ligands was investigated 11, 40, 44 in which the therapeutic efficacy of IRDye700DX-based PSMA ligands was described in two preclinical studies 11, 40. In addition, Overchuk *et al*. developed and tested a bacteriochlorophyll-based PSMA-targeted photosensitizer, which allowed for potent and precise image-guided photodynamic treatment of PSMA-expressing tumors 44. The first study to describe tPDT with multimodal tracers for PCa detection, resection and ablation was performed by Harmatys *et al*. 45. They developed the theranostic agent LC-pyro, consisting of a PSMA-binding motif and porphyrin photosensitizer capable of fluorescence imaging, PDT, and ^64^Cu chelation for PET imaging. Next, Lütje *et al*. developed the PSMA-targeting murine antibody [^111^In]In-DTPA-D2B-IRDye700DX 46. The main advantage of mAbs is the high absolute tumor uptake, caused by their high affinity and long circulatory half-life. Nonetheless, accumulation of mAbs in solid tumors can take up to days and the mAb D2B is a murine IgG that should at least be humanized before clinical translation is feasible. Compared to mAbs, small molecule multimodal tracers may show better tumor penetration and more rapid blood clearance, enabling injection at the day of surgery 15, 39, 47.

In conclusion, the ligand structure optimization performed in our study showed that addition of the fluorophore (IRDye700DX), and additional negative charges in the linker or chelator part of the ligand, significantly improves tumor targeting. We demonstrated that our best ligands - N064, N140 and N142 - had a similar overall performance and were able to visualize PMSA-positive tumors using both radionuclide and fluorescence imaging in a pre-clinical model and on fresh PCa tissue samples. The optimized multimodal PSMA ligands developed in this study could have potential for four applications; preoperative visualization of tumors using SPECT/CT, radio-guided surgery for intraoperative tumor localization, NIRF-guided surgical resection, and ablation of tumor tissue via tPDT in areas where tumor remnants cannot be removed surgically due to proximity to other vital structures. Together, these optimized multimodal ligands could revolutionize image-guided resection and intraoperative tPDT of PCa tumors.

## Supplementary Material

Supplementary figures and tables.

## Figures and Tables

**Table 1:** Name, structure, affinity, lipophilicity, membrane bound fractions and internalized fractions of the multimodal PSMA ligands.

General structure 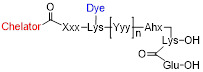							
**Name**	**Chelator**	**Xxx**	**[Yyy]_n_**	**IRDye 700DX**	**Membrane bound fraction**	**Internalized fraction**	**LogD**
N025	DOTAGA		*D*-Phe	No	1.7 ±0.01	2.0 ± 0.08	-2.8 ± 0.08
N046	DOTAGA		*D*-Phe	Yes	0.8 ± 0.06	2.0 ± 0.39	-2.4 ± 0.03
N057b	DOTAGA		*D*-Phe-Glu-Glu	No	1.4 ± 0.05	2.0 ± 0.06	-2.8 ± 0.04
N064	DOTAGA		*D*-Phe-Glu-Glu	Yes	0.6 ± 0.05	4.6 ± 0.31	-2.6 ± 0.06
NJ26	DOTAGA	PEG_4_	*D*-Phe-Glu-Glu	Yes	0.5 ± 0.05	1.8 ± 0.03	-2.4 ± 0.13
NJ27	DOTA	PEG_4_	*D*-Phe-Glu-Glu	Yes	0.3 ± 0.04	1.1 ± 0.05	-2.3 ± 0.11
N111	DOTAGA		PEG_4_-*D*-Phe-Glu-Glu	Yes	0.4 ± 0.05	0.9 ± 0.06	-2.4 ± 0.15
N122	DOTA	Glu	*D*-Phe-Glu-Glu	Yes	0.6 ± 0.01	1.4 ± 0.20	-2.8 ± 0.06
N140	DOTA		*D*-Phe-Glu-Glu-Glu	Yes	0.2 ± 0.07	2.5 ± 0.07	-2.4 ± 0.04
N142	DOTAGA		*D*-Phe-Glu-Glu-Glu	Yes	0.2 ± 0.06	4.5 ± 0.20	-2.3 ± 0.05
N143	DOTAGA		Glu-Glu-Glu	Yes	0.5 ± 0.20	3.6 ± 0.22	-2.4 ± 0.10
N144	DOTAGA		Glu-Glu	Yes	0.3 ± 0.03	2.5 ± 0.05	-2.4 ± 0.01
N064inc*	DOTAGA		*D*-Phe-Glu-Glu	Yes	0.1 ± 0.07	0.05 ± 0.03	-
**Name**	**IC_50_ (nM)**		**K_d_ (nM)**				
N064	42.1 (95% CI: 22.6-78.1)		6.9 ± 3.5				
PSMA-617	52.7 (95% CI: 21.1-131.1)		15 ± 1.1				

Abbreviations: PEG_4_: poly(ethylene glycol)4; *D*-Phe: *D*-Phenylalanine; Glu: Glutamic acid. Data are presented as mean ± SD. *N064inc: Incomplete, lacking the glutamic acid in the PSMA-binding motif.

## Abbreviations

2-PMPA: 2-(phosphonomethyl)pentane-1,5-dioic acid
*d*-Phe: d-phenylalanine
NIRF: Near infrared fluorescence imaging
PCa: Prostate cancer
PSMA: Prostate specific membrane antigen
tPDT: targeted photodynamic therapy
^111^In: Indium-111
