# Supplementary material for: Photosensitizer-based multimodal PSMA-targeting ligands for intraoperative detection of prostate cancer
Source: Theranostics. 2021 Jan 1;11(4):1527–41. doi: 10.7150/thno.52166 (PMC7778589; doi:10.7150/thno.52166)
Supplement: Supplementary file 1 — Supplementary figures and tables. [file thnov11p1527s1.pdf]

Supplementary Materials and Methods

Synthesis of the PSMA binding motif

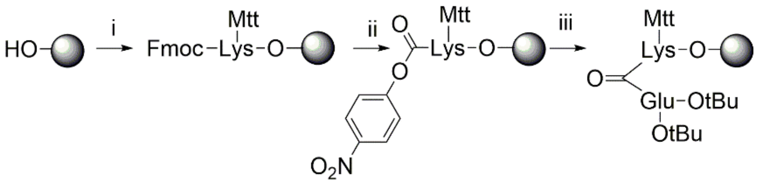

Figure. S1. Synthesis of PSMA binding motif.

i) Wang resin (1 eq., 1.0 mmol/g, 1.00 g) was swollen in 10 mL DMF for 10 minutes. Fmoc-Lys(Mtt)-OH (3 eq., 3 mmol, 1.87 g), 4-dimethylaminopyridine (1 eq., 1 mmol, 122.2 mg), HOBt (3.6 eq., 3.6 mmol, 1M in DMF) and DIPCDI (3.3 eq., 3.3 mmol, 1M in DMF) were added to the resin and mixed on a bench roller for 20 hours. The reagents were removed from the resin by vacuum filtration. The resin was washed with DMF (3x10 mL) and DCM (3x10 mL). The Fmoc-loading was determined to be 0.5 mmol/g. Next, the resin was capped with a solution of pyridine (0.34 mL/g resin) and benzoyl chloride (0.34 mL/g resin) in DCM for 1 hour.

ii) The resin was washed with DCM (3x10 mL) and DMF (3x10 mL) and after Fmoc removal (20% piperidine in DMF, 3x6 min), DIPEA (0.52 mL, 3 eq., 3 mmol,), 4-nitrophenyl chloroformate (2 eq., 2.0 mmol, 402 mg) in 2 mL DCM were added to the H-Lys(Mtt)-resin (1eq, 0.5 mmol/g, 2 g) and the resin was agitated for 1 hour. Consecutively a Kaiser test was performed to check for completion (*1*).

iii) Glutamic acid di-*tert*-butyl ester hydrochloride (3 eq., 3 mmol, 887.4 mg) and DIPEA (4 eq., 4 mmol, 0.70 mL) in DCM were added to the resin and the mixture was agitated for 1 hour. The resin was washed with DCM (3x10 mL) and DMF (3x10 mL).

General synthesis of the ligands

Mtt deprotection: Resin was treated with 1.8% TFA in $CHCl_3$ for 4-5 times during 5 minutes until the filtrate was not yellow anymore. Per 100 mg of resin 3 mL of TFA/ $CHCl_3$ solution was used. The deprotection was checked with UV-Vis and mass spectrometry.

DIPCDI coupling of protected amino acids: Fmoc-protected amino acid (3.0 eq.), 1-hydroxybenzotriazole hydrate (HOBt, 1M in DMF, 3.6 eq.), N, N'-Diisopropylcarbodiimide (DIPCDI, 1M in DMF, 3.3 eq.) were added to the resin and agitated until the Kaiser test was negative (~45 minutes) after which the resin was washed with DMF (3x10 mL).

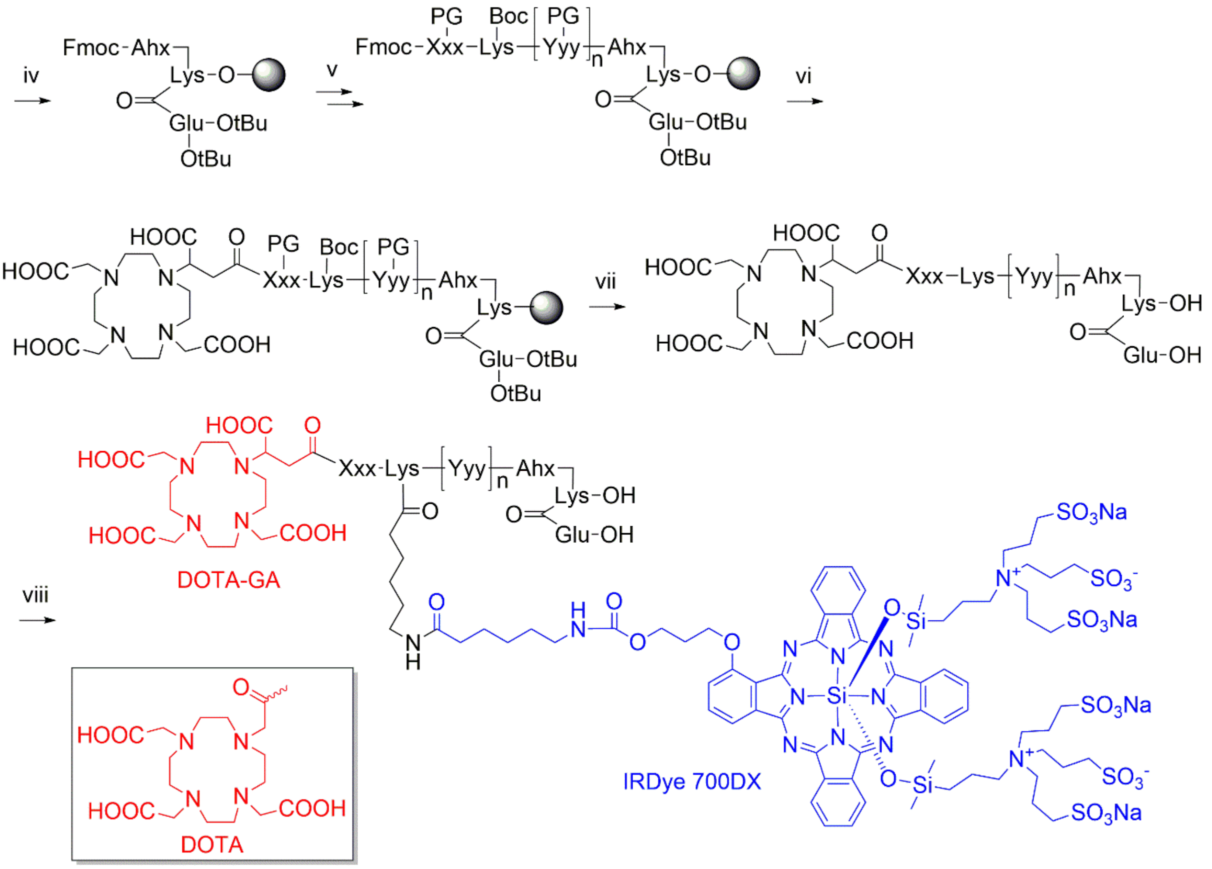

Figure. S2. Synthesis of ligands (shown for DOTAGA as a chelator).

HATU coupling of protected amino acids: Fmoc-protected amino acid (3.0 eq.), 1-hydroxybenztriazole hydrate (HOBt, 3.6 eq.), N, N, N′, N′-Tetramethyl-O-(1H-benzotriazol-1-yl)uronium hexafluorophosphate (HBTU, 2.9 eq.) and N, N'-diisopropylethylamine (DIPEA, 6 eq.) were dissolved in DMF. The solution was pre-activated for 2 minutes before it was added to the resin. The mixture was agitated until the Kaiser test was negative (~1.5 hrs.) after which the resin was washed with DMF (3x10 mL) and DCM (3x10 mL).

Fmoc deprotection: The resin was treated with 20% piperidine in DMF 3x6 minutes. The product was washed with DMF (3x10 mL).

DOTAGA or DOTA coupling: DIPEA (2 eq.) and DOTAGA anhydride or DOTA-OSu were added to the resin in NMP and mixed on a bench roller at room temperature or stirred at 70°C respectively for 6-8 hrs.

Resin cleavage: All peptides were cleaved from the resin with trifluoroacetic acid/H_2O (95:5, v/v) for two hours after which the resin was filtered off and the peptide was precipitated in diethyl ether. After drying in air the crude peptide was lyophilized from water.

Conjugation with IRDye700DX: Peptide was dissolved in phosphate buffer (0.25 M, pH 8) after which the dye OSu ester (1.0 eq. in dry DMF) was added and shaken at rt for 4-6 hrs. The product was purified directly by preparative HPLC.

Analytical HPLC: Compounds were analyzed on a Shimadzu LC-20A Prominence system with a dual UV-Vis detector (Shimadzu, 's Hertogenbosch, The Netherlands) equipped with a C18 Gemini-NX column, 150 × 3 mm, particle size 3 μm (Phenomenex, Utrecht, The Netherlands) Solvent A was 0.1% trifluoroacetic acid (TFA) in H_2O and solvent B was 0.1% TFA in acetonitrile (MeCN). A gradient of 5-100% acetonitrile (30 min.) was applied.

Preparative HPLC: All compounds were purified on a Shimadzu dual-pump LC-20A Prominence system (Shimadzu, 's Hertogenbosch, The Netherlands) equipped with a C18 Gemini-NX column, 150 × 10 mm, particle size 10 μm (Phenomenex, Utrecht, The

Netherlands), applying a gradient of 20-70% methanol in triethylammonium acetate buffer (10 mM, pH 7) for all IRDye containing compounds or a gradient of 5-100% acetonitrile in water (0.1% TFA) for all others.

Competitive binding assays

Scatchard analysis was performed to determine the dissociation constant (Kd) of the N064 ligand. LS174T-PSMA-positive cells were cultured to confluence in 6-wells plates, washed with 2 ml PBS and incubated for 4 h on ice with increasing concentrations of ^{111}In-labeled ligand (0.03 – 30 nM) in 1 ml binding buffer (RPMI 1640 containing 0.5% w/v BSA). Non-specific binding was determined by coincubation with 1 µM PSMA-617. After incubation, cells were washed with 2 ml PBS twice and lysed with 1.5 ml 0.1 M NaOH, which causes detachment and lysis of the cells from the 6-wells plate. Cell lysis was collected from the plate and the cell-associated activity was measured in a γ-counter. The specific binding (total binding – nonspecific binding) was plotted against the bound/free ratio. To determine PSMA antigen density per cell and the Kd of multimodal ligands, data were analyzed by linear regression using GraphPad Prism software.

The 50% inhibitory concentration (IC50) of PSMA-N064 was determined using PSMA-expressing LS174T-PSMA cells in a competitive binding assay. The LS174T-PSMA cells were cultured to confluency in 6-wells plates, followed by washing with 2 ml PBS and incubation on ice for 2 h in 1 mL of binding buffer (RPMI 1640 containing 0.5% w/v BSA) with 50,000 cpm of 111In-labeled ligand and a series of increasing concentrations (0.01-300 nM) of unlabeled PSMA-N064. After incubation, cells were washed with 2 ml PBS twice and lysed with 1.5 ml 0.1 M NaOH, which causes detachment and lysis of the cells from the 6-wells plate. Cell lysis was collected from the plate and the cell-associated activity was measured in a γ-counter and IC50 values were calculated using GraphPad Prism software.

Supplementary Results

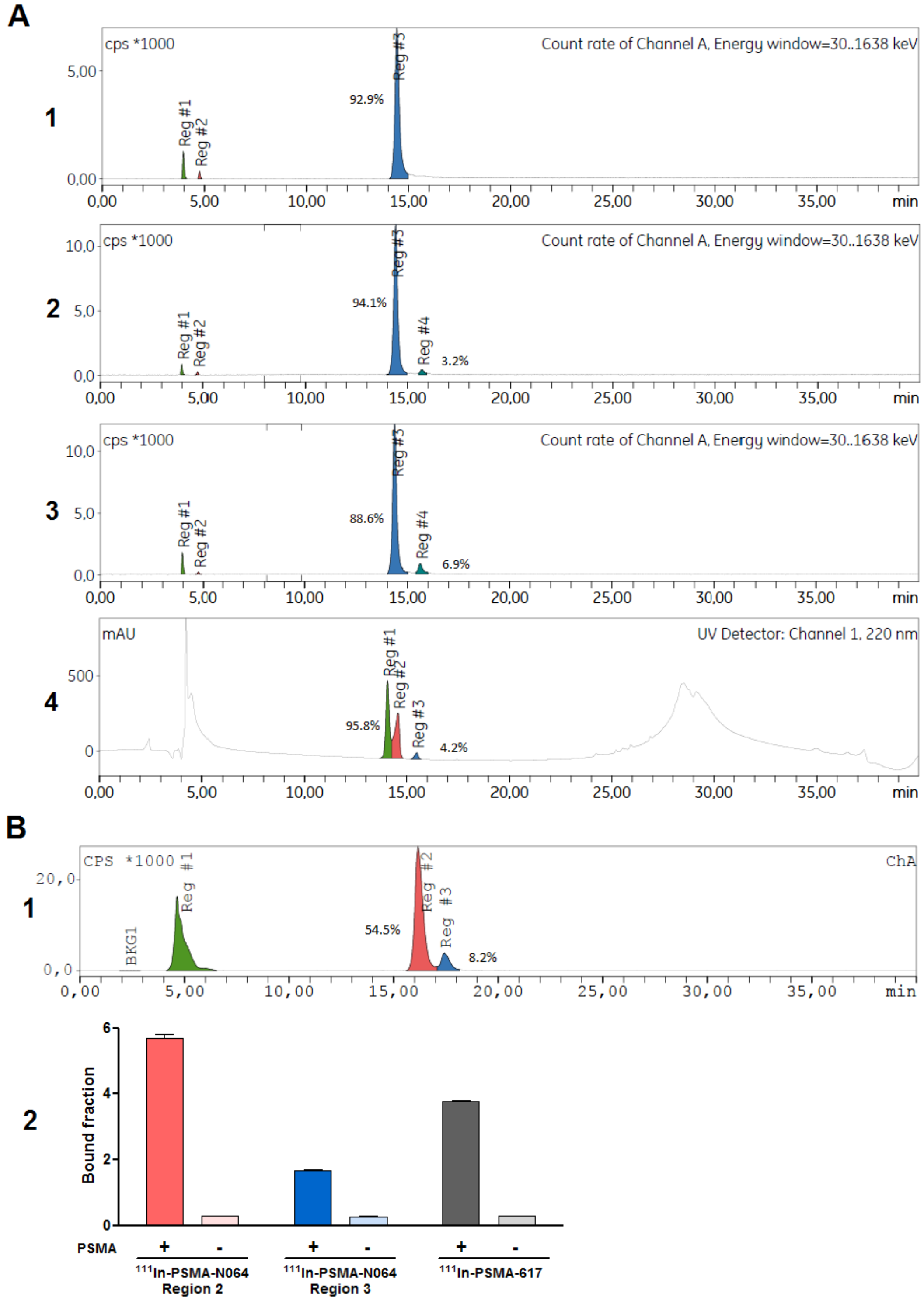

Figure. S3. Stability of ^{111}In-PSMA-N064 during radiolabeling at pH 5.5, 45 °C. (A) Radionuclide HPLC chromatogram of ^{111}In-PSMA-N064 labeled with $^{111}InCl_3$ for 10 min [1], 20 min [2], 30 min [3], and fluorescence HPLC chromatogram labeled with $^{111}InCl_3$ for 30 min [4] in 2-(N-morpholino)ethanesulfonic acid (MES) buffer, 5 MBq/µg, pH5.5, 45 °C. **(B)** [1] Radioactivity HPLC chromatogram of ^{111}In-PSMA-N064 labeled with $^{111}InCl_3$ for 30 min. Peaks 2 and 3 were collected for a subsequent binding assay. [2] PSMA-bound fraction of peak 1, peak 2 and ^{111}In-PSMA-617 (reference compound) in LS174T-PSMA and LS174T wildtype cells *in vitro.*

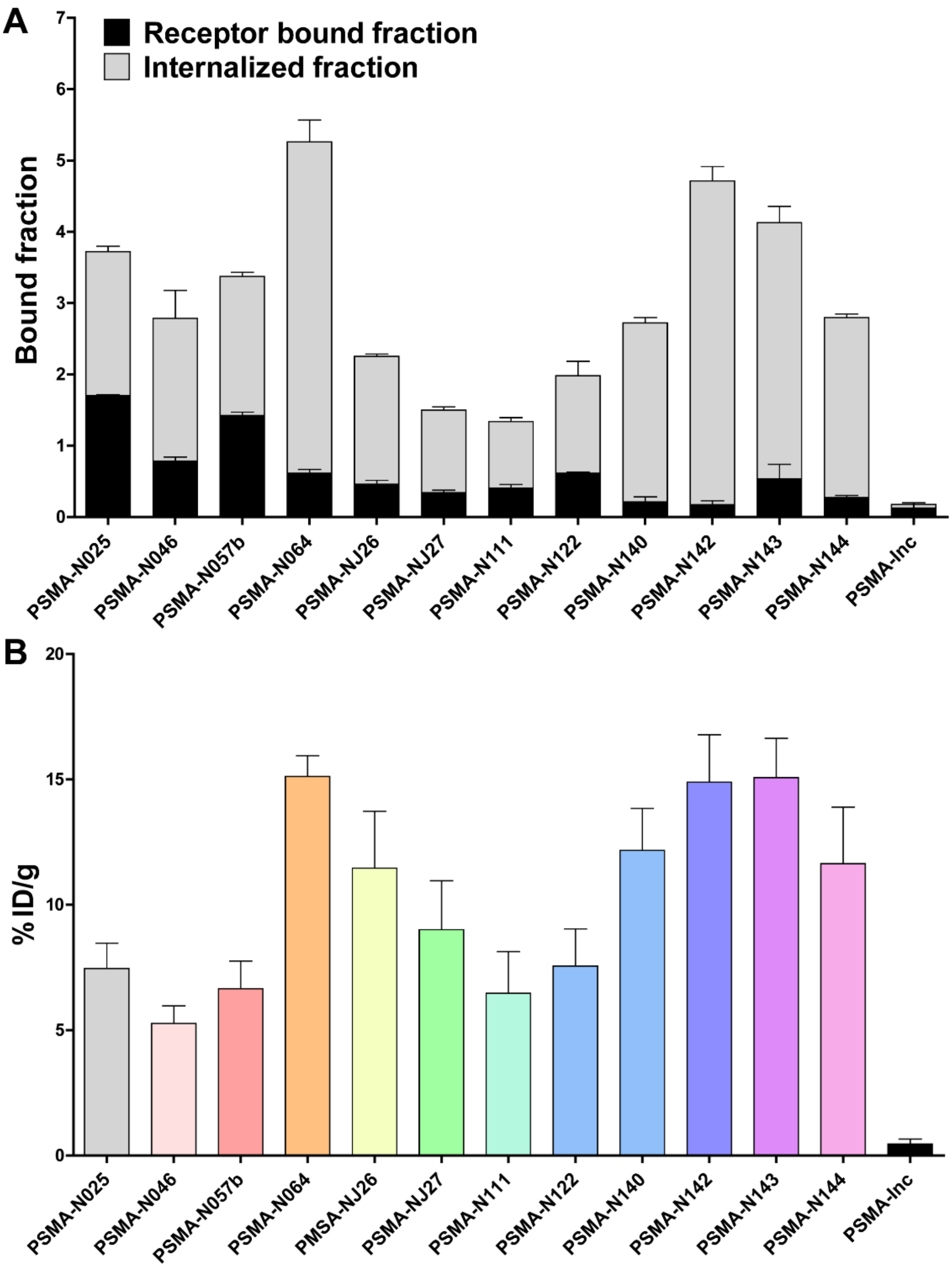

Figure. S4. Uptake of ^{111}In-labeled multimodal ligands in PSMA-positive cells *in vitro* and *in vivo*. (A) PSMA-receptor bound and internalized fraction of 12 multimodal ligands in PSMA-positive LS174T-PSMA cells *in vitro*. **(B)** PSMA-positive tumor uptake of ligands (n = 5mice/group, 0.3 nmol/mouse, 2 hrs p.i., 10 MBq ^{111}In/mouse) in mice bearing LS174T-PSMA xenografts.

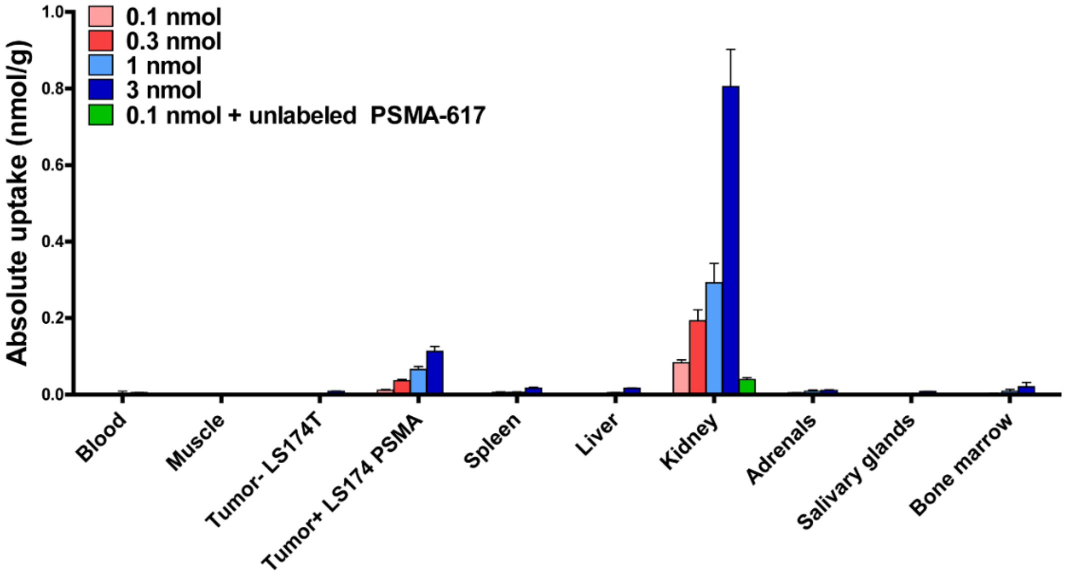

Figure. S5. Absolute uptake of ^{111}In-PSMA-N064 ligand. Absolute uptake of ^{111}In-PSMA-N064 (n = 5mice/group, 0.1-3 nmol/mouse, 2 hrs p.i., 1 MBq/mouse) in mice bearing LS174T-PSMA and LS174T xenografts, including effect of PSMA-617 co-injection (10 nmol).

Table S1. Dose and time optimization of ^{111}In-PSMA-N064 in mice bearing LS174T-PSMA and LS174T xenografts.

	0.1 nmol	0.3 nmol	1 nmol	3 nmol	0.1 + 100 nmol*	1 hr	2 hrs	4 hrs	24 hrs
**Biodistribution**									
Blood	0.2 ± 0.2	0.4 ± 0.3	0.4 ± 0.6	0.2 ± 0.1	0.2 ± 0.1	0.7 ± 0.2	0.5 ± 0.2	0.1 ± 0.1	0.1 ± 0.1
Muscle	0.4 ± 0.4	0.5 ± 0.3	0.2 ± 0.1	0.1 ± 0.1	0.1 ± 0.1	0.4 ± 0.2	0.2 ± 0.1	0.1 ± 0.1	0.1 ± 0.1
Tumor- LS174T	0.4 ± 0.1	0.5 ± 0.1	0.3 ± 0.1	0.4 ± 0.1	0.4 ± 0.2	0.8 ± 0.2	0.6 ± 0.2	0.3 ± 0.1	0.3 ± 0.1
Tumor+ LS174T-PSMA	11.5 ± 2	12.2 ± 1.1	6.6 ± 0.8	4.6 ± 0.6	1.2 ± 0.1	9.9 ± 1.4	13.1 ± 2.4	8 ± 0.5	4.6 ± 1.9
Heart	0.4 ± 0.1	0.4 ± 0.1	0.2 ± 0.1	0.2 ± 0.1	0.3 ± 0.1	0.7 ± 0.2	0.6 ± 0.1	0.3 ± 0.1	0.2 ± 0.1
Lung	0.5 ± 0.1	0.5 ± 0.1	0.3 ± 0.1	0.3 ± 0.1	0.2 ± 0.1	1 ± 0.2	0.8 ± 0.1	0.3 ± 0.1	0.1 ± 0.1
Spleen	1.6 ± 0.4	1.9 ± 0.5	0.6 ± 0.1	0.7 ± 0.1	0.5 ± 0.1	3.3 ± 1.1	2 ± 0.5	0.8 ± 0.2	0.4 ± 0.1
Pancreas	0.3 ± 0.1	0.3 ± 0.1	0.2 ± 0.1	0.2 ± 0.1	0.1 ± 0.1	0.4 ± 0.1	0.3 ± 0.1	0.2 ± 0.1	0.1 ± 0.1
Liver	0.8 ± 0.1	1 ± 0.2	0.5 ± 0.1	0.7 ± 0.1	0.7 ± 0.2	1 ± 0.2	1.1 ± 0.2	0.7 ± 0.2	0.6 ± 0.1
Stomach	0.3 ± 0.1	0.5 ± 0.2	0.2 ± 0.1	0.2 ± 0.1	0.4 ± 0.6	0.6 ± 0.2	0.5 ± 0.1	0.2 ± 0.1	0.2 ± 0.1
Kidney	82.9 ± 7.8	64.4 ± 9.7	29.3 ± 5.1	32.2 ± 4	39.5 ± 5.3	72.1 ± 9.5	77.3 ± 10.4	50.7 ± 4.1	16.6 ± 1.7
Adrenals	1.6 ± 0.4	1.5 ± 0.3	0.9 ± 0.4	0.5 ± 0.1	0.5 ± 0.2	2.2 ± 0.6	2.4 ± 0.3	0.8 ± 0.4	0.3 ± 0.2
Duodenum	0.3 ± 0.1	0.3 ± 0.1	0.2 ± 0.1	0.2 ± 0.1	0.2 ± 0.1	0.5 ± 0.1	0.4 ± 0.2	0.2 ± 0.1	0.2 ± 0.1
Prostate	0.5 ± 0.2	0.6 ± 0.2	0.3 ± 0.3	0.4 ± 0.3	0.2 ± 0.1	1.3 ± 1	1.5 ± 1.6	0.3 ± 0.2	0.2 ± 0.1
Salivary glands	0.6 ± 0.1	0.6 ± 0.2	0.3 ± 0.1	0.4 ± 0.1	0.4 ± 0.1	0.8 ± 0.2	0.6 ± 0.1	0.3 ± 0.1	0.3 ± 0.1
Bone marrow	1.6 ± 2.3	1 ± 0.5	0.9 ± 0.6	0.9 ± 0.5	0.5 ± 0.2	0.7 ± 0.2	0.3 ± 0.5	0.2 ± 0.2	0.4 ± 0.4
Bone	0.7 ± 0.2	0.8 ± 0.1	0.5 ± 0.2	0.6 ± 0.1	0.4 ± 0.1	0.7 ± 0.2	0.7 ± 0.3	0.4 ± 0.1	0.3 ± 0.1
**Tumor/Organ ratios**									
Tumor/Blood	51.4 ± 15.6	47.4 ± 19.9	45.3 ± 34.6	23.4 ± 3.3	13.3 ± 4.6	17.1 ± 5.6	33.8 ± 6.4	112.1 ± 19	391.2 ± 187.8
Tumor/Kidney	0.2 ± 0.1	0.2 ± 0.1	0.3 ± 0.1	0.2 ± 0.1	0.1 ± 0.1	0.2 ± 0.1	0.2 ± 0.1	0.2 ± 0.1	0.3 ± 0.1
Tumor/Muscle	53.8 ± 46.2	37.5 ± 19.3	66.8 ± 25.5	59.1 ± 13.3	16.3 ± 6.1	34.8 ± 10.7	75.9 ± 27	114.2 ± 35.4	110 ± 52.1
Tumor/Negative tumor	36.3 ± 8.7	30.1 ± 7.5	25.4 ± 6	13.3 ± 2.5	3.9 ± 1.2	13.8 ± 2.8	23.5 ± 2.9	28 ± 6.4	19.8 ± 4.2
Tumor/Spleen	7.7 ± 3	6.9 ± 2.1	11.4 ± 2.9	6.7 ± 0.8	2.9 ± 0.5	3.3 ± 1	7 ± 1.7	11.7 ± 2.5	13.7 ± 6.8
Tumor/Liver	16.1 ± 4.2	13.9 ± 2.9	13.8 ± 1.5	6.8 ± 1	1.8 ± 0.4	10.2 ± 1.8	12.3 ± 0.4	13 ± 2.6	8.6 ± 3.4
Tumor/Salivary gland	22.5 ± 5	24.4 ± 6.5	28 ± 6	14.6 ± 1.3	4 ± 0.9	12.8 ± 2.6	22.3 ± 3	30.4 ± 6.6	19.8 ± 10.9
Tumor/Prostate	32.9 ± 21.1	23 ± 7.2	44.7 ± 36.1	16.7 ± 9	8.8 ± 3.8	12.7 ± 10.5	16.3 ± 8.5	37.3 ± 12.3	42.5 ± 19.8

Data are presented as Mean ± SD, *100 nmol unlabeled PSMA-617 added.

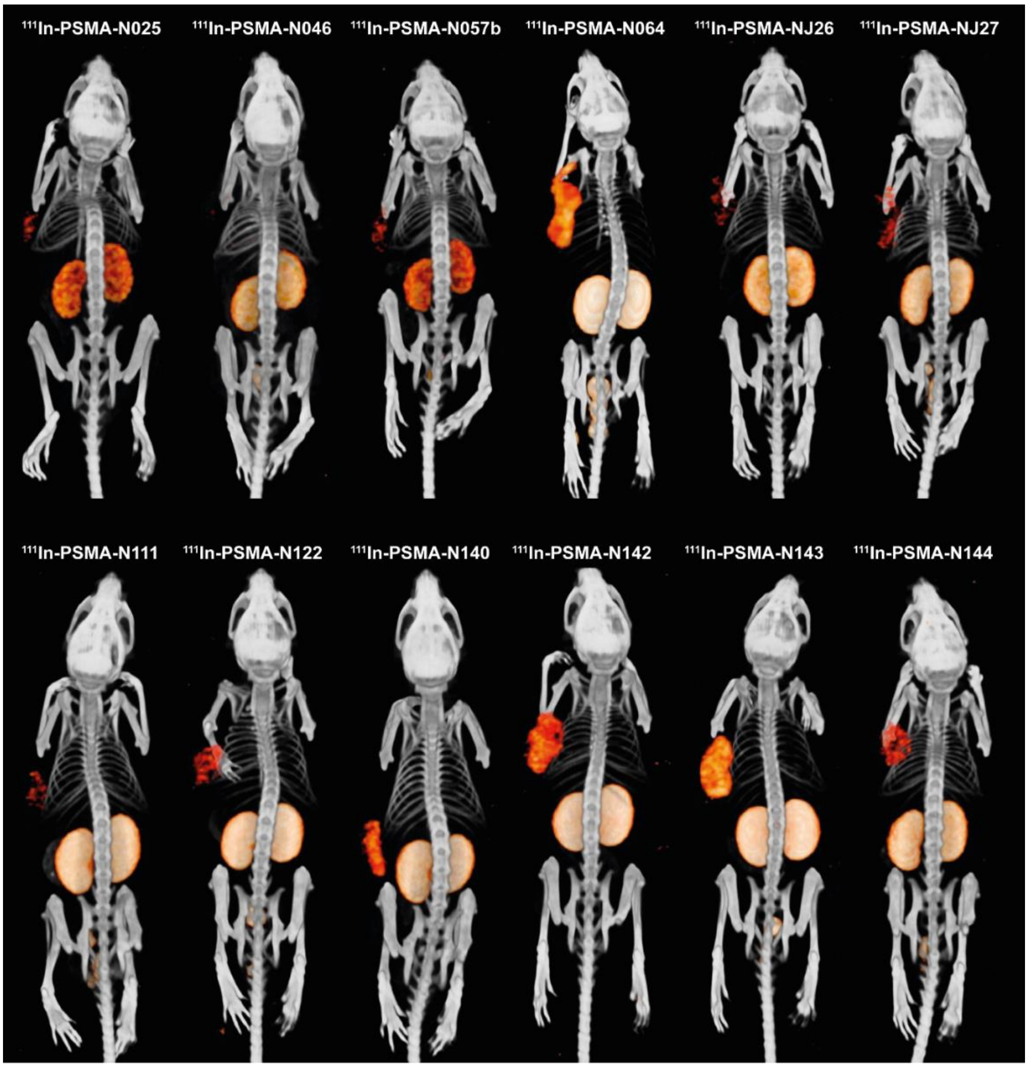

Figure. S6. Same scale µSPECT/CT images of 12 multimodal PSMA-ligands. Same scale µSPECT/CT images of mice with s.c. LS174T-PSMA (left) and wildtype LS174T (right) tumors after i.v. injection of 12 ^{111}In-labeled multimodal ligands (0.3 nmol, 10 MBq/mouse, 2 h p.i.).

Table S2. Biodistribution of ^{111}In-labeled multimodal ligands in mice bearing LS174T-PSMA and LS174T xenografts

	PSMA -N025	PSMA -N046	PSMA -N57b	PSMA -N064	PSMA -NJ26	PSMA -NJ27	PSMA -N111	PSMA -N122	PSMA -N140	PSMA -N142	PSMA -N143	PSMA -N144
**Biodistribution**												
Blood	0.1 ± 0.1	0.7 ± 0.9	0.1 ± 0.1	0.5 ± 0.2	0.3 ± 0.1	0.2 ± 0.2	0.2 ± 0.1	0.6 ± 0.1	0.6 ± 0.2	0.4 ± 0.1	0.3 ± 0.1	0.2 ± 0.1
Muscle	0.1 ± 0.1	0.2 ± 0.1	0.1 ± 0.1	0.3 ± 0.2	0.2 ± 0.1	0.1 ± 0.1	0.1 ± 0.1	0.2 ± 0.1	0.4 ± 0.4	0.2 ± 0.1	0.3 ± 0.1	0.2 ± 0.1
Tumor- LS174T	0.2 ± 0.1	0.4 ± 0.1	0.1 ± 0.1	0.7 ± 0.2	0.4 ± 0.2	0.4 ± 0.1	0.4 ± 0.2	0.5 ± 0.2	0.8 ± 0.2	0.5 ± 0.1	0.5 ± 0.1	0.5 ± 0.2
Tumor+ LS174T-PSMA	7.5 ± 1.1	4.3 ± 0.6	6.7 ± 1.2	15.2 ± 0.9	11.5 ± 2.3	9.1 ± 2	6.5 ± 1.7	7.6 ± 1.5	12.2 ± 1.7	14.9 ± 1.9	15.1 ± 1.6	11.7 ± 2.3
Heart	0.1 ± 0.1	0.3 ± 0.1	0.1 ± 0.1	0.6 ± 0.1	0.3 ± 0.1	0.3 ± 0.1	0.3 ± 0.1	0.5 ± 0.1	0.5 ± 0.1	0.6 ± 0.1	0.5 ± 0.1	0.3 ± 0.1
Lung	0.3 ± 0.4	0.4 ± 0.1	0.2 ± 0.1	0.8 ± 0.1	0.5 ± 0.2	0.3 ± 0.1	0.4 ± 0.1	1 ± 0.3	0.9 ± 0.2	0.8 ± 0.1	0.7 ± 0.1	0.4 ± 0.1
Spleen	0.4 ± 0.3	1.1 ± 0.3	0.2 ± 0.1	2 ± 0.2	0.9 ± 0.3	0.6 ± 0.3	1.3 ± 0.5	1.6 ± 0.3	2.1 ± 0.3	2.2 ± 0.7	3.1 ± 1	1.1 ± 0.4
Pancreas	0.1 ± 0.1	0.2 ± 0.1	0.1 ± 0.1	0.5 ± 0.4	0.2 ± 0.1	0.2 ± 0.1	0.2 ± 0.1	0.3 ± 0.1	0.4 ± 0.1	0.4 ± 0.2	0.4 ± 0.1	0.2 ± 0.1
Liver	0.3 ± 0.3	0.8 ± 0.1	0.1 ± 0.1	0.9 ± 0.4	0.6 ± 0.2	0.6 ± 0.1	0.5 ± 0.1	1 ± 0.2	0.9 ± 0.1	1.3 ± 0.1	1 ± 0.2	0.6 ± 0.1
Stomach	0.2 ± 0.1	0.3 ± 0.1	0.1 ± 0.1	0.6 ± 0.1	0.3 ± 0.1	0.2 ± 0.1	0.3 ± 0.1	0.4 ± 0.1	0.7 ± 0.2	0.5 ± 0.1	0.5 ± 0.1	0.3 ± 0.1
Kidney	22 ± 9.6	31.9 ± 2.9	15.7 ± 3.3	85.5 ± 5.1	59.9 ± 11.7	51.4 ± 6.5	59 ± 7.4	72.5 ± 12.7	63.3 ± 7.6	122.4 ± 10.2	112.8 ± 14.1	70.3 ± 3.7
Adrenals	0.8 ± 0.5	1.2 ± 0.5	0.5 ± 0.1	1.5 ± 0.3	1.5 ± 0.4	0.7 ± 0.1	0.9 ± 0.2	1.7 ± 0.6	1.7 ± 0.6	1.8 ± 0.5	2.5 ± 1.8	1.3 ± 0.2
Duodenum	0.1 ± 0.1	0.3 ± 0.1	0.1 ± 0.1	0.4 ± 0.1	0.3 ± 0.1	0.2 ± 0.1	0.2 ± 0.1	0.4 ± 0.1	0.5 ± 0.1	0.4 ± 0.1	0.6 ± 0.5	0.3 ± 0.1
Prostate	0.5 ± 0.9	0.9 ± 0.8	0.5 ± 0.8	0.4 ± 0.2	0.5 ± 0.2	0.3 ± 0.1	0.5 ± 0.2	0.4 ± 0.1	1.3 ± 1.4	0.4 ± 0.1	1.4 ± 2.3	0.5 ± 0.2
Salivary glands	0.1 ± 0.1	0.4 ± 0.1	0.1 ± 0.1	0.6 ± 0.1	0.4 ± 0.2	0.3 ± 0.1	0.4 ± 0.1	0.5 ± 0.1	0.8 ± 0.2	0.6 ± 0.2	0.6 ± 0.1	0.4 ± 0.1
Bone marrow	0.2 ± 0.2	0.2 ± 0.1	0.1 ± 0.1	0.3 ± 0.2	0.4 ± 0.3	0.4 ± 0.3	0.3 ± 0.3	0.4 ± 0.2	0.4 ± 0.1	0.3 ± 0.2	0.4 ± 0.2	0.3 ± 0.1
Bone	0.2 ± 0.2	0.4 ± 0.1	0.1 ± 0.1	1 ± 0.2	0.2 ± 0.1	0.2 ± 0.1	0.3 ± 0.1	0.7 ± 0.2	0.9 ± 0.4	1.2 ± 0.2	1.2 ± 0.2	0.5 ± 0.1
**Tumor/ Organ ratios**												
Tumor/Blood	127.9 ± 58	14.9 ± 8	128.4 ± 66.5	35.5 ± 8.1	49.1 ± 7.3	62.6 ± 24.9	41.1 ± 6.8	13.9 ± 1.9	21 ± 4.1	48.2 ± 12.5	56 ± 7.9	71.9 ± 11.8
Tumor/Kidney	0.4 ± 0.2	0.2 ± 0.1	0.5 ± 0.1	0.2 ± 0.1	0.2 ± 0.1	0.2 ± 0.1	0.2 ± 0.1	0.2 ± 0.1	0.2 ± 0.1	0.2 ± 0.1	0.2 ± 0.1	0.2 ± 0.1
Tumor/Muscle	245.1 ± 111.7	39.3 ± 6.1	234.2 ± 72	71.8 ± 28	116.4 ± 27.4	106.4 ± 30.8	71.9 ± 28.9	48.4 ± 10.1	47.9 ± 23.5	86.9 ± 19.9	72.3 ± 20.1	110 ± 22.9
Tumor/Negative tumor	72.3 ± 14.7	10.8 ± 1.2	89.5 ± 21.2	25.9 ± 7.3	31.7 ± 9	27.1 ± 4.4	24.1 ± 10.3	17.8 ± 4.8	18.1 ± 5.5	30.5 ± 4.7	36.7 ± 6.5	28.8 ± 5.2
Tumor/Spleen	24.4 ± 10.3	4.4 ± 1	43.9 ± 22.7	7.8 ± 0.8	14.2 ± 4.6	17.3 ± 3.1	5.7 ± 2.4	5.2 ± 1.2	6.1 ± 1.4	7.7 ± 2.4	5.2 ± 1.2	12.3 ± 4.1
Tumor/Liver	95.6 ± 15.5	6.1 ± 0.9	112.2 ± 14.4	21.9 ± 16.5	21.3 ± 3.2	15.9 ± 2.1	13.7 ± 3.8	8.1 ± 1.3	14.1 ± 3	11.9 ± 1.6	15.2 ± 1.7	21.3 ± 3.1
Tumor/Salivary gland	88.2 ± 37.9	12.5 ± 2.1	100.8 ± 26	26.3 ± 5	35 ± 7.3	33.2 ± 4.7	21.5 ± 6.8	18.6 ± 5	17.5 ± 2.7	27.8 ± 6.5	28.8 ± 2.8	30.3 ± 7.5
Tumor/Prostate	61.4 ± 53.6	10.7 ± 8.9	58 ± 32.7	48.4 ± 21.5	29.4 ± 8.7	32.7 ± 7.7	17.2 ± 11	21.5 ± 6.8	16.2 ± 9.3	45.6 ± 4.7	32.9 ± 18.7	32 ± 10.3

Data are presented as Mean ± SD.

Table S3. Age, Gleason score before and after surgery and signal-to-noise ratio per patient

**Patient number**	Age (years)	Gleason score before surgery*	Gleason score after surgey**	SNR: directly adjacent tissue****	SNR: contralateral healthy prostate
**1**	58	4+3 = 7	4+3 = 7	2.1	2.2
**2**	58	4+5 = 9	4+5 = 9	-	7.3
**3**	56	3+4 = 7	3+4 = 7	2.1	3.5
**4**	63	4+5 = 9	3+5(+4) = 8	3.6	4.9
**5**	72	5+4 = 9	4+5 = 9	2.7	2.5
**6**	70	3+4 = 7	4+3 = 7	2.5	3.7
**7**	54	3+3 = 6	3+4 = 7	2.5	2.5
**8**	55	5+4 = 9	4+3 = 7	3.4	2.0
**9*** **	68	3+4 = 7	4+3 = 7		
**10*** **	67	3+4 = 7	3+3 = 6		

* Gleason score of the tumor as determined by a pathologist using biopsies taken before surgery.
** Gleason score of the tumor as determined by a pathologist after surgical removal of the prostate.
*** No malignancy in biopsies taken after radical prostatectomy, patients excluded.
**** SNR: Signal-to-noise ratio

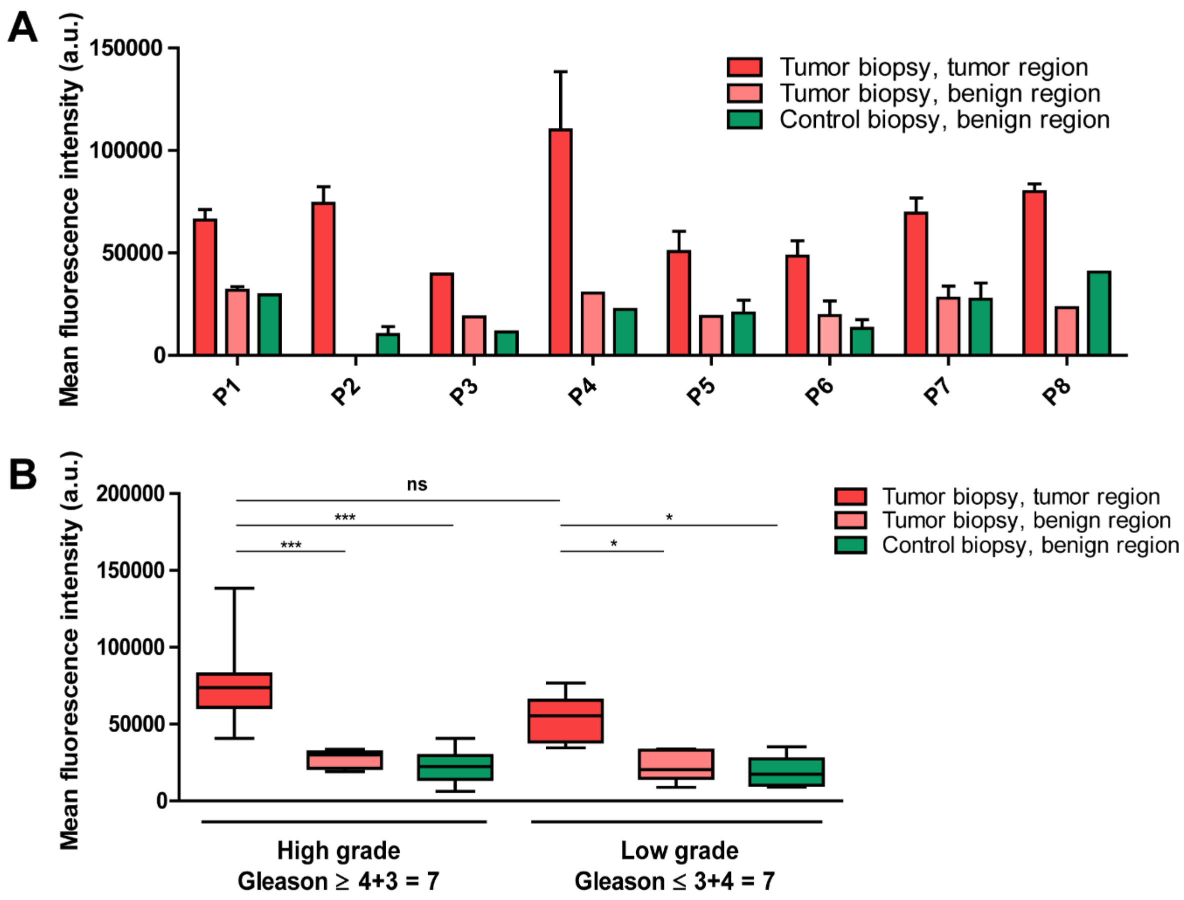

Figure. S7. Quantification of ^{111}In-PSMA-N064 and ^{111}In-PSMA-N140 incubated prostate cancer biopsies taken during radical prostatectomy. (A) Mean fluorescence intensity per patient (P1-8), based on Odyssey fluorescence images. **(B)** Mean fluorescence intensity of low grade (Gleason ≤ 3+4 = 7) and low grade (Gleason ≥ 4+3 = 7) biopsies, as determined by a pathologist using biopsies taken before surgery. Tumor regions within the tumor biopsy were compared to fluorescence intensity in normal regions within the tumor biopsy and normal regions in the control biopsy, as defined by a pathologist. * <0.01, *** <0.001, ns = not significant.

Figure S8. Structure, HPLC chromatogram, ESI-MS and MALDI-ToF spectra of multimodal ligands.

N025

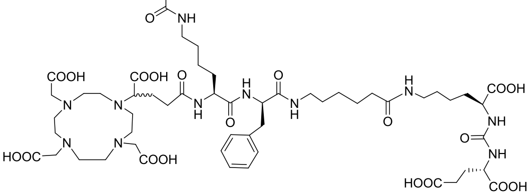

Chemical Formula: $C_{54}H_{85}N_{11}O_{20}$
Exact Mass: 1207.60
m/z: 1207.60 (100.0%), 1208.60 (58.4%), 1209.60 (16.7%)

N046

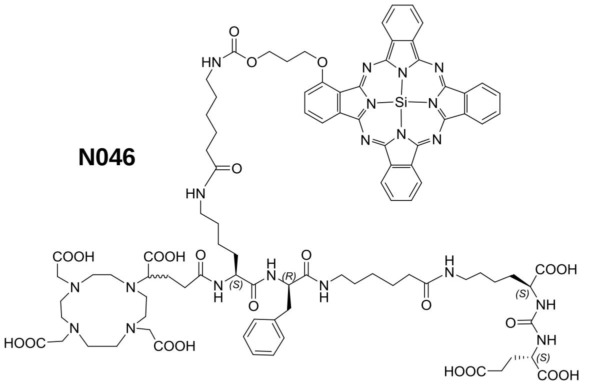

Chemical Formula: $C_{94}H_{114}N_{20}O_{23}Si$
m/z: 1919.82 (100.0%), 1918.81 (98.4%), 1920.82 (42.1%), 1921.82 (11.5%)

N057b

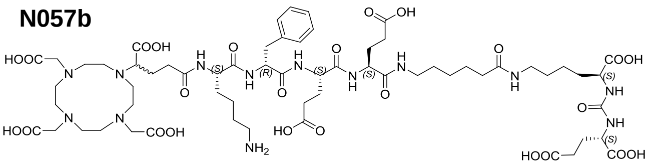

Chemical Formula: $C_{62}H_{97}N_{13}O_{25}$
Exact Mass: 1423.67
m/z: 1423.67 (100.0%), 1424.68 (67.1%), 1425.68 (22.1%)

N064

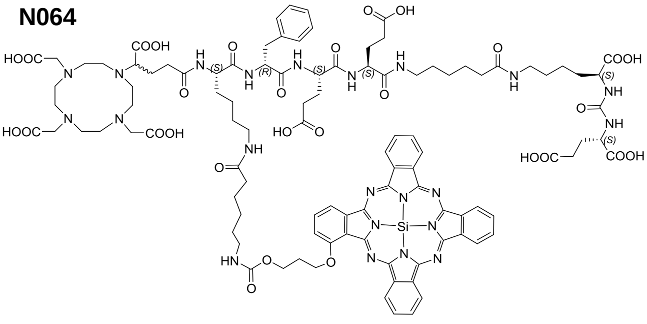

Chemical Formula: $C_{104}H_{128}N_{22}O_{29}Si$
m/z: 2177.90 (100.0%), 2176.90 (88.9%), 2178.91 (38.0%), 2179.91 (18.9%)

NJ26

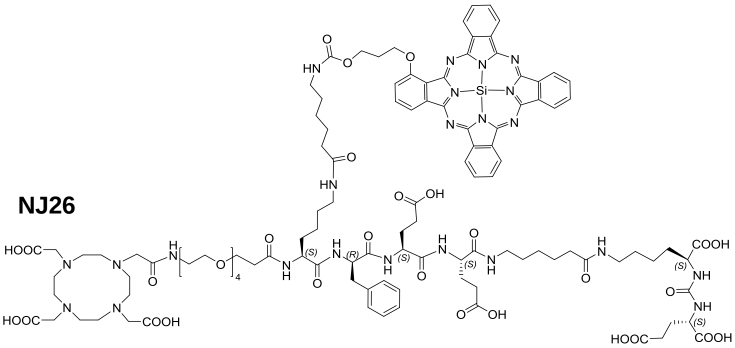

Chemical Formula: $C_{112}H_{145}N_{23}O_{32}Si$
m/z: 2353.02 (100.0%), 2352.02 (82.6%), 2354.03 (35.3%), 2354.03 (24.7%)

NJ27

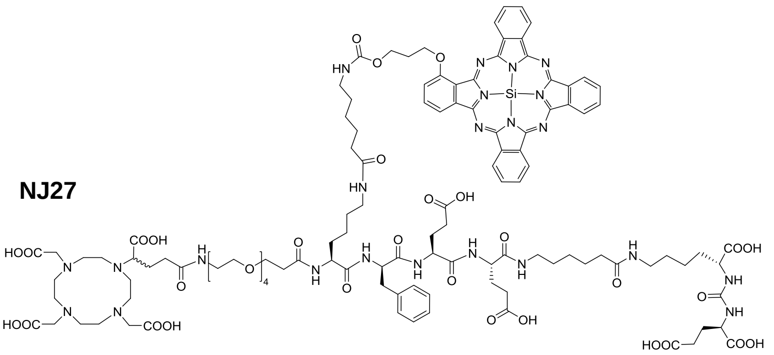

Chemical Formula: $C_{115}H_{149}N_{23}O_{34}Si$
m/z: 2425.04 (100.0%), 2424.04 (80.4%), 2426.05 (61.6%), 2427.05 (21.2%)

N111

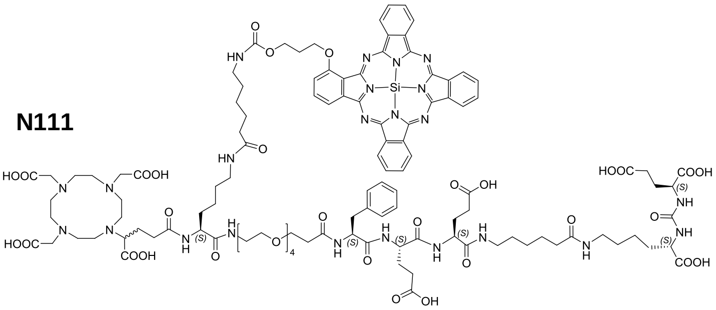

Chemical Formula: $C_{115}H_{149}N_{23}O_{34}Si$
m/z: 2425.04 (100.0%), 2424.04 (80.4%), 2426.05 (61.6%), 2427.05 (21.2%)

N122

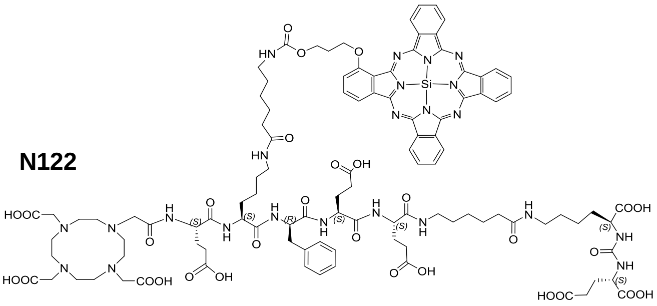

Chemical Formula: $C_{106}H_{131}N_{23}O_{30}Si$
m/z: 2234.92 (100.0%), 2233.92 (87.2%), 2235.93 (56.8%), 2236.93 (11.1%)

N140

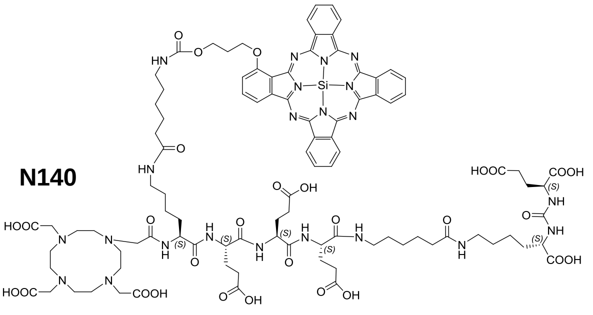

Chemical Formula: $C_{97}H_{122}N_{22}O_{29}Si$
m/z: 2087.86 (100.0%), 2086.85 (95.3%), 2088.86 (40.8%), 2089.86 (17.2%)

N142

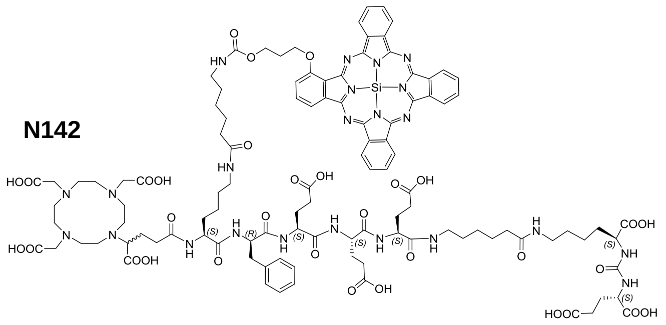

Chemical Formula: $C_{109}H_{135}N_{23}O_{32}Si$
m/z: 2306.94 (100.0%), 2305.94 (84.8%), 2307.95 (58.4%), 2308.95 (12.6%)

N143

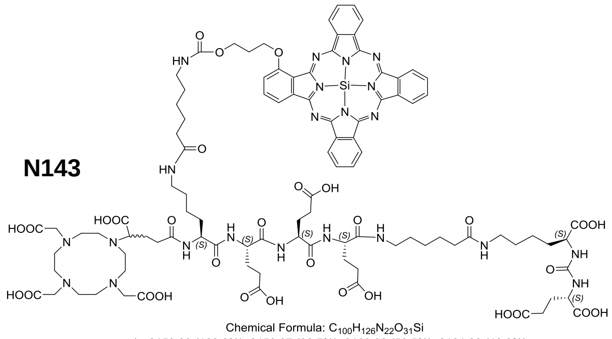

Chemical Formula: $C_{100}H_{126}N_{22}O_{31}Si$
m/z: 2159.88 (100.0%), 2158.87 (92.5%), 2160.88 (53.5%), 2161.88 (10.8%)

N144

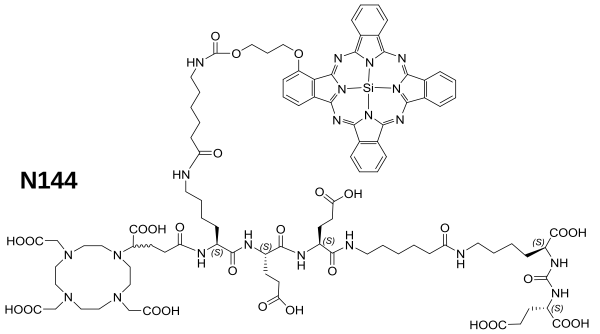

Chemical Formula: $C_{95}H_{119}N_{21}O_{28}Si$
m/z: 2030.83 (100.0%), 2029.83 (97.3%), 2031.84 (41.6%), 2032.84 (11.4%)

PSMA-N025

HPLC chromatogram

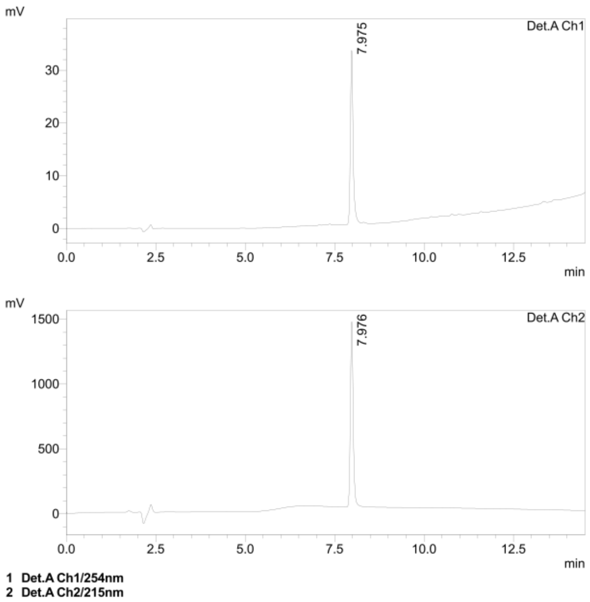

ESI-ion trap spectrum

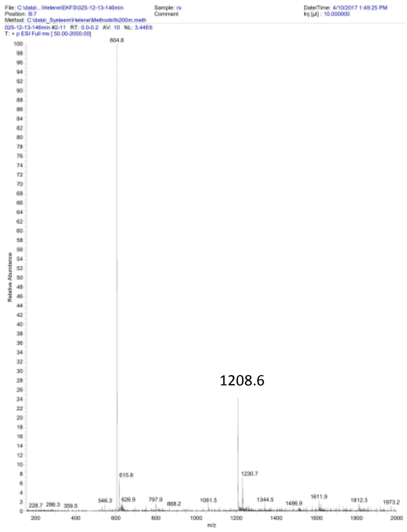

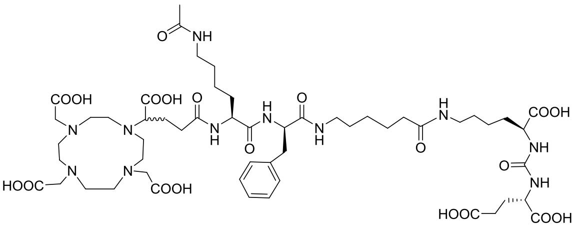

Chemical Formula: $C_{54}H_{85}N_{11}O_{20}$
Exact Mass: 1207.60
m/z: 1207.60 (100.0%), 1208.60 (58.4%), 1209.60 (16.7%)

PSMA-N046

HPLC chromatogram

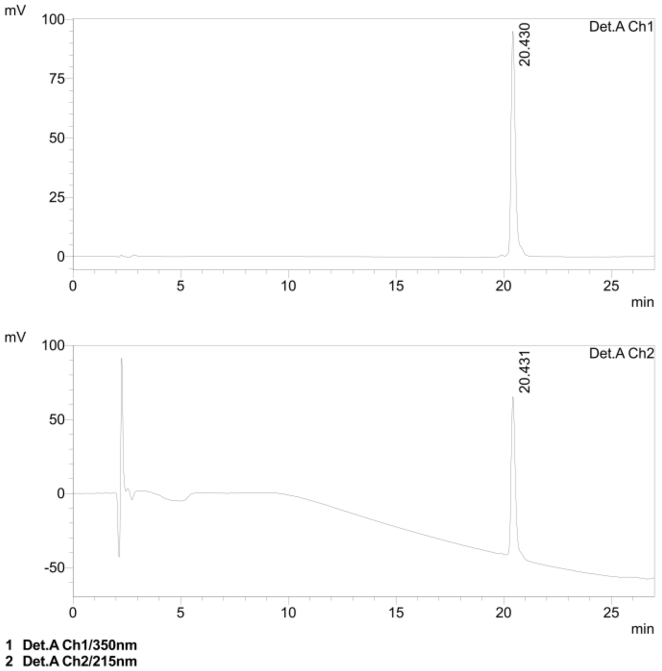

MALDI-ToF spectrum with matrix **HCCA**

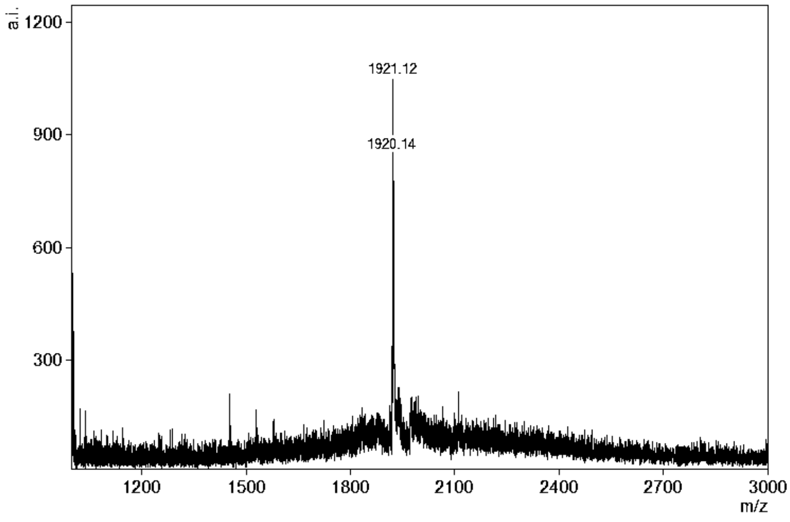

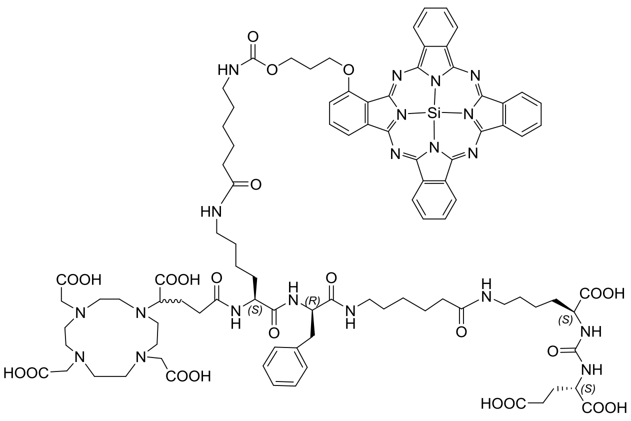

Chemical Formula: $C_{94}H_{114}N_{20}O_{23}Si$

m/z: 1919.82 (100.0%), 1918.81 (98.4%), 1920.82 (42.1%), 1921.82 (11.5%)

PSMA-N057b

HPLC chromatogram

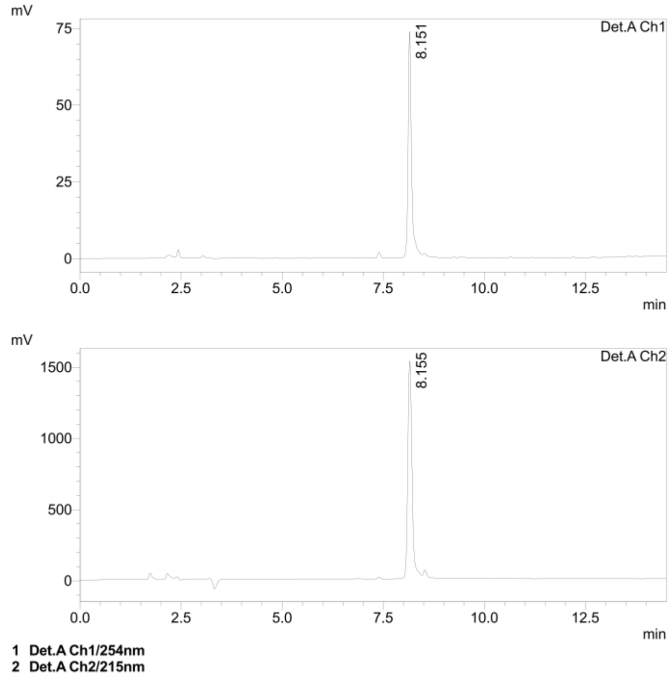

ESI-ion trap spectrum

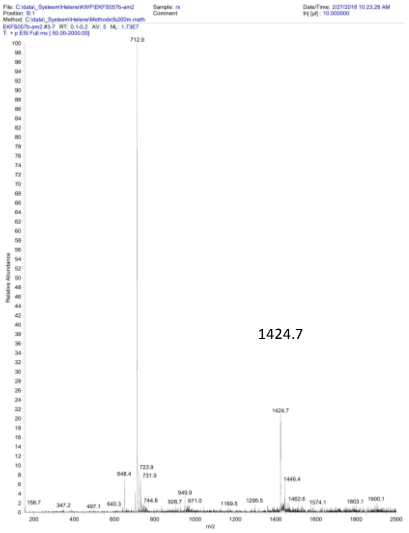

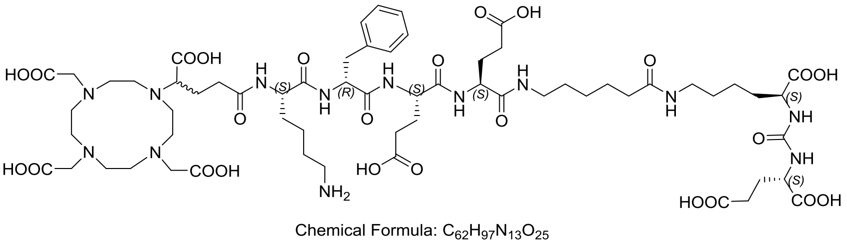

Chemical Formula: $C_{62}H_{97}N_{13}O_{25}$
Exact Mass: 1423.67
m/z: 1423.67 (100.0%), 1424.68 (67.1%), 1425.68 (22.1%)

PSMA-N064

HPLC chromatogram

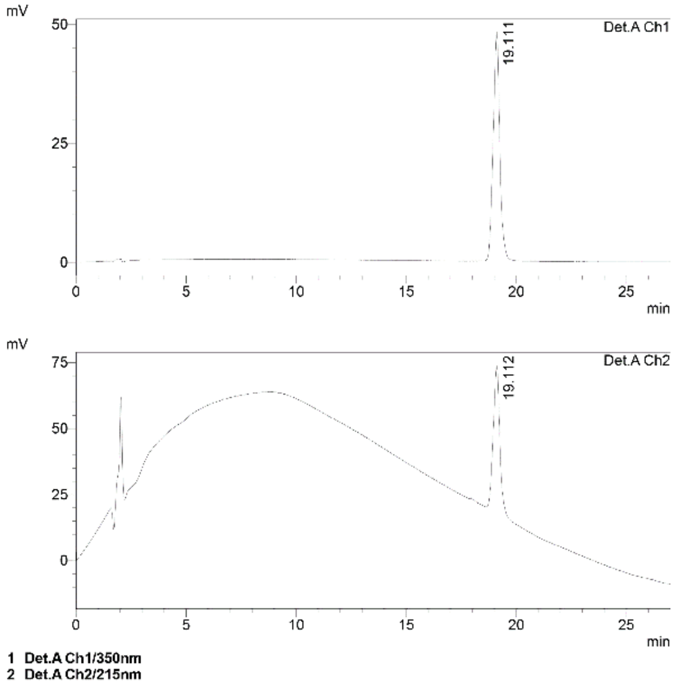

MALDI-ToF spectrum with matrix **HCCA**

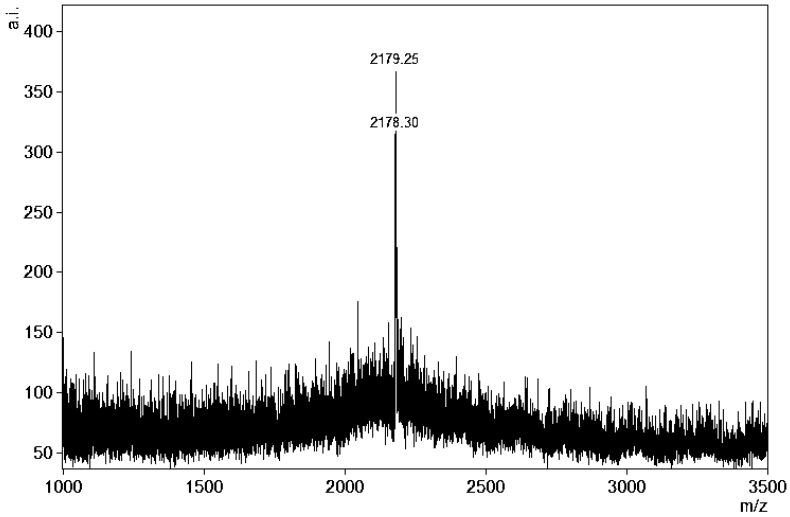

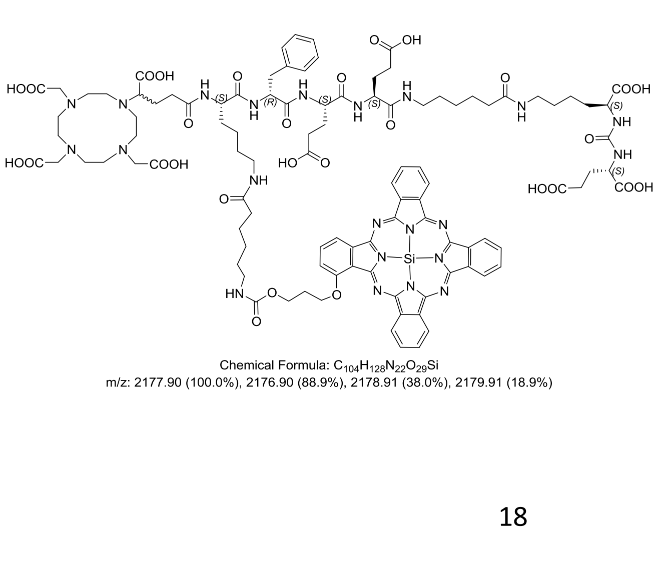

Chemical Formula: $C_{104}H_{128}N_{22}O_{29}Si$
m/z: 2177.90 (100.0%), 2176.90 (88.9%), 2178.91 (38.0%), 2179.91 (18.9%)

PSMA-NJ26

HPLC chromatogram

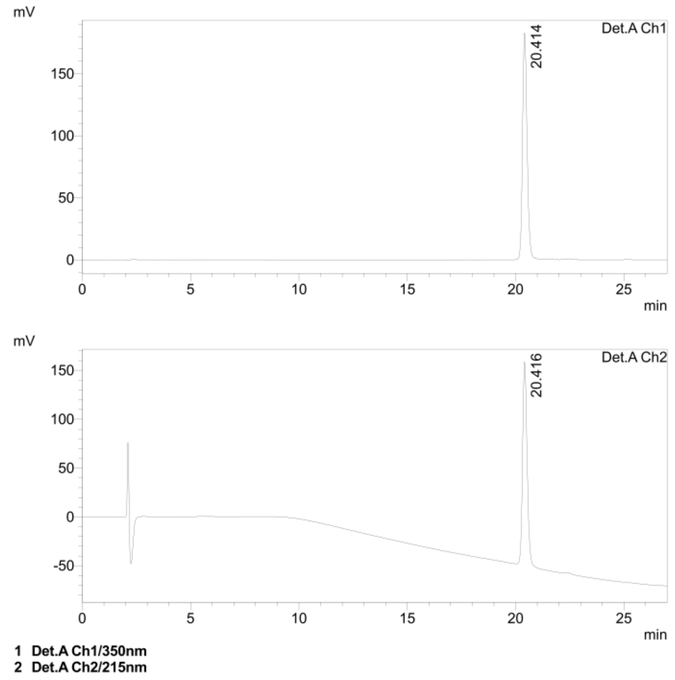

MALDI-ToF spectrum with matrix **HCCA**

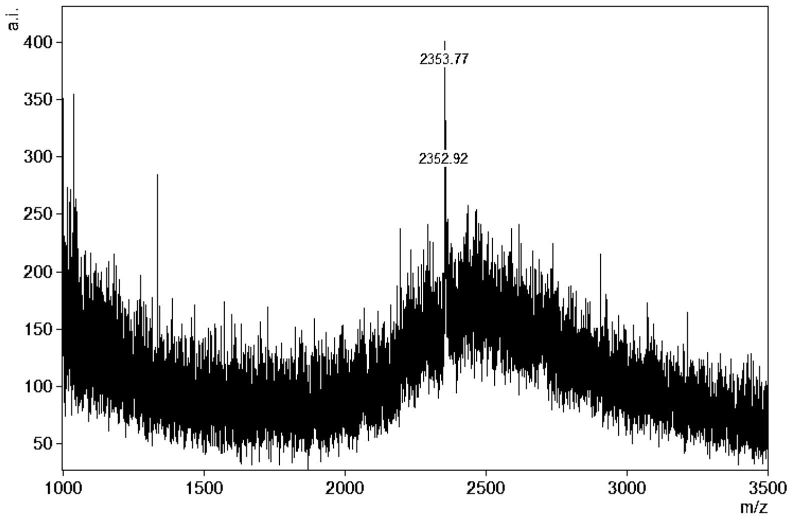

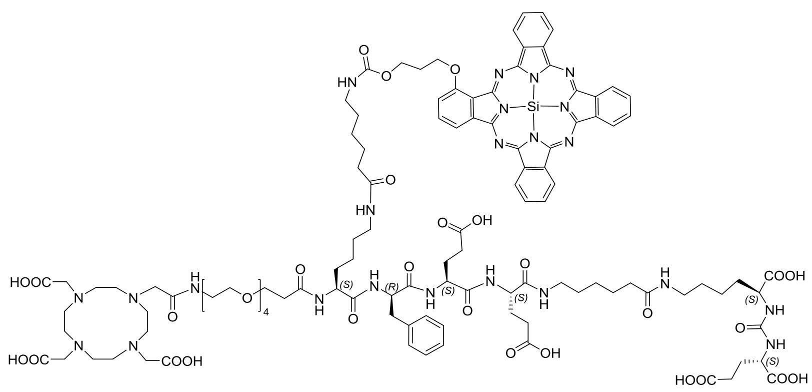

Chemical Formula: $C_{112}H_{145}N_{23}O_{32}Si$
m/z: 2353.02 (100.0%), 2352.02 (82.6%), 2354.03 (35.3%), 2354.03 (24.7%)

PSMA-NJ027

HPLC chromatogram

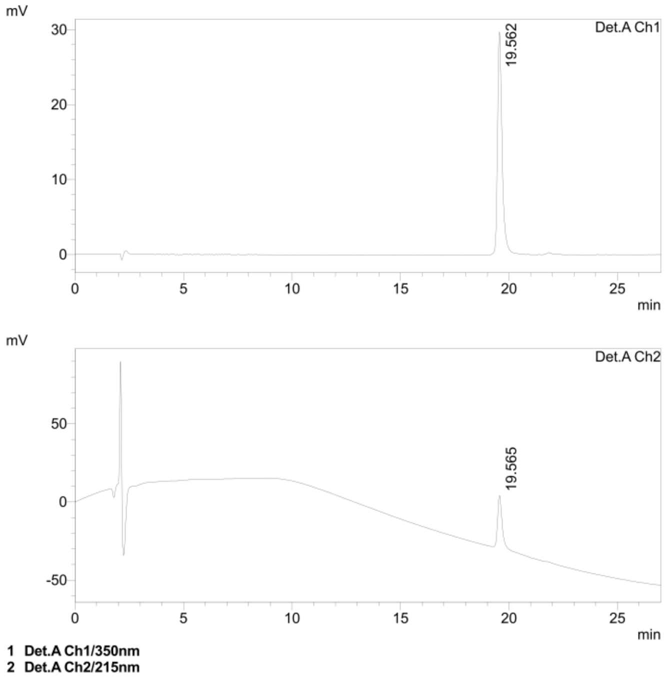

MALDI-ToF spectrum with matrix **HCCA**

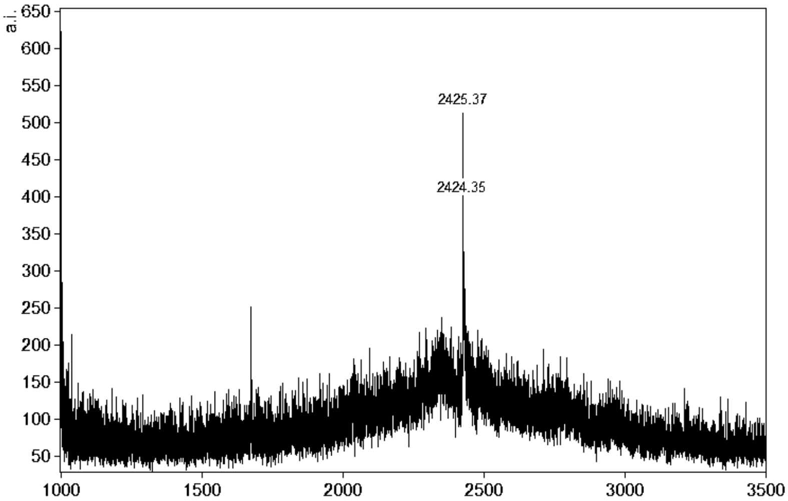

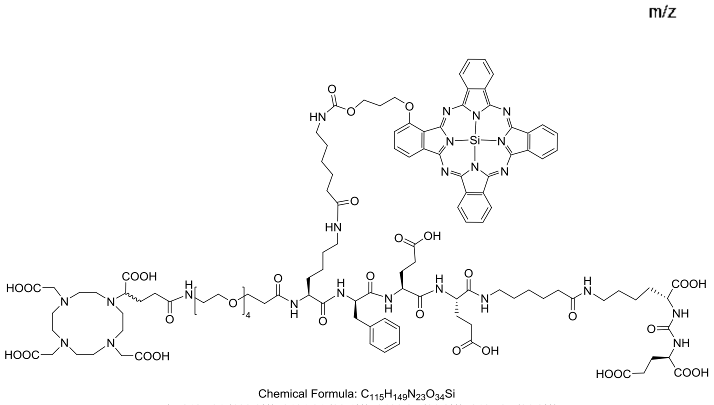

Chemical Formula: $C_{115}H_{149}N_{23}O_{34}Si$
m/z: 2425.04 (100.0%), 2424.04 (80.4%), 2426.05 (61.6%), 2427.05 (21.2%)

PSMA-N111

HPLC chromatogram

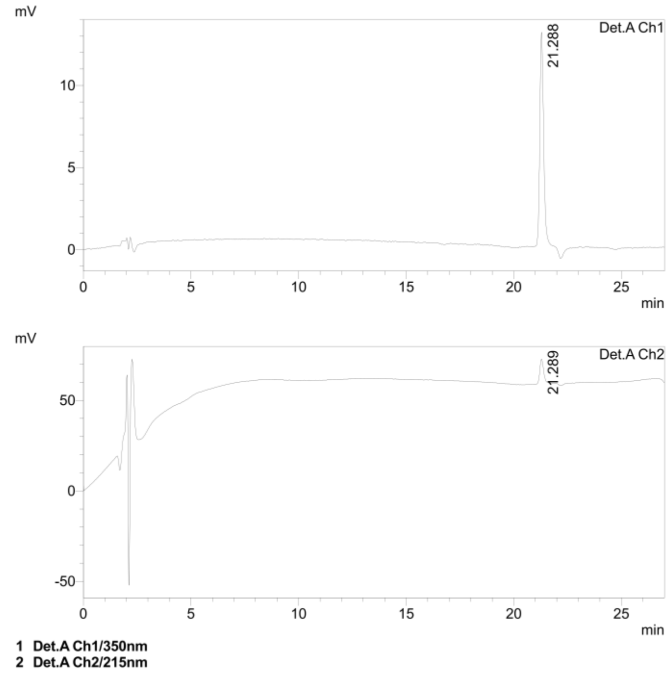

MALDI-ToF spectrum with matrix **HCCA**

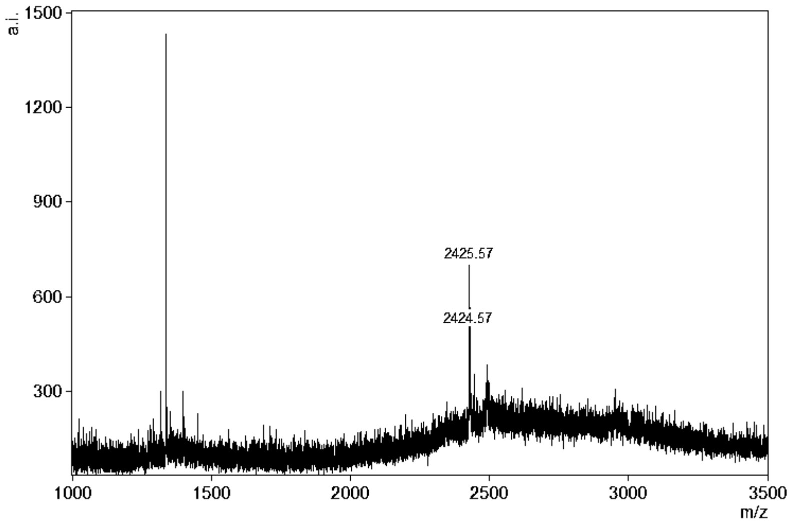

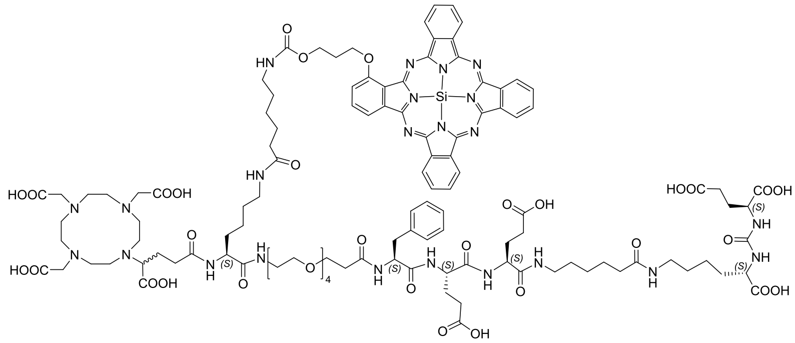

Chemical Formula: $C_{115}H_{149}N_{23}O_{34}Si$
m/z: 2425.04 (100.0%), 2424.04 (80.4%), 2426.05 (61.6%), 2427.05 (21.2%)

PSMA-N122

HPLC chromatogram

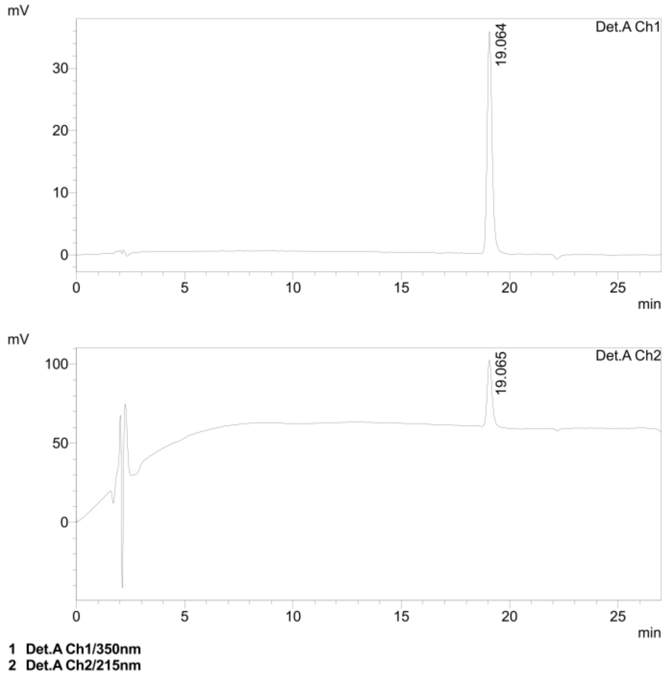

MALDI-ToF spectrum with matrix **HCCA**

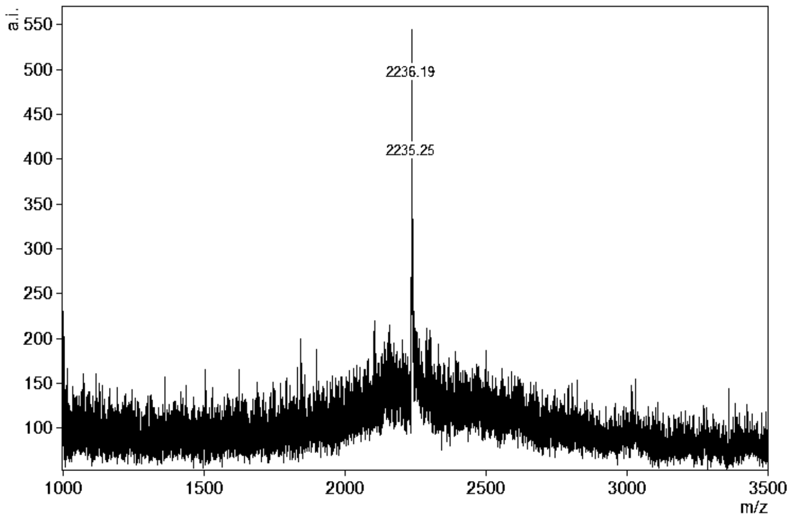

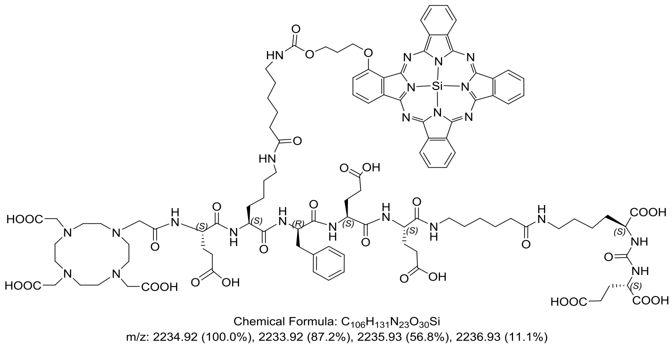

Chemical Formula: $C_{106}H_{131}N_{23}O_{30}Si$

m/z: 2234.92 (100.0%), 2233.92 (87.2%), 2235.93 (56.8%), 2236.93 (11.1%)

PSMA-N140

HPLC chromatogram

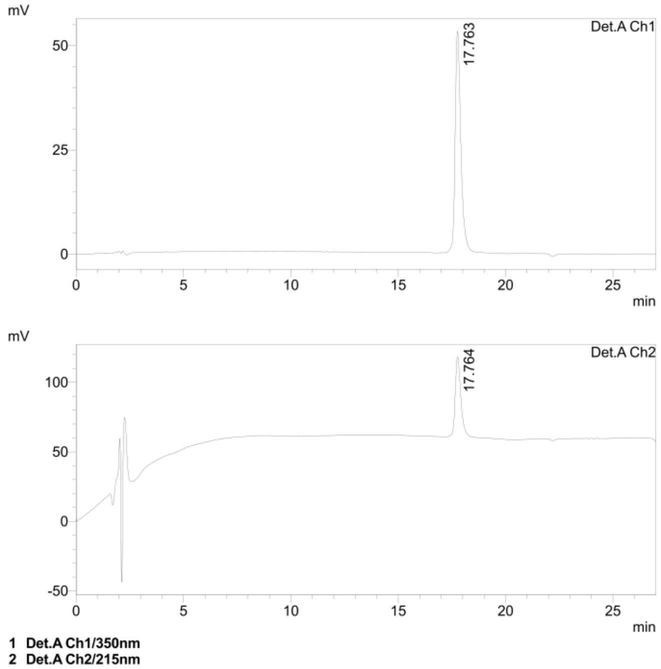

MALDI-ToF spectrum with matrix HCCA

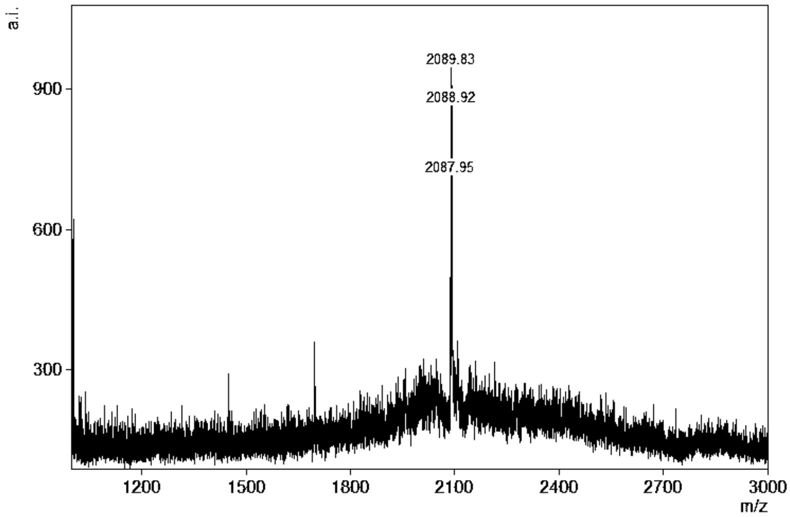

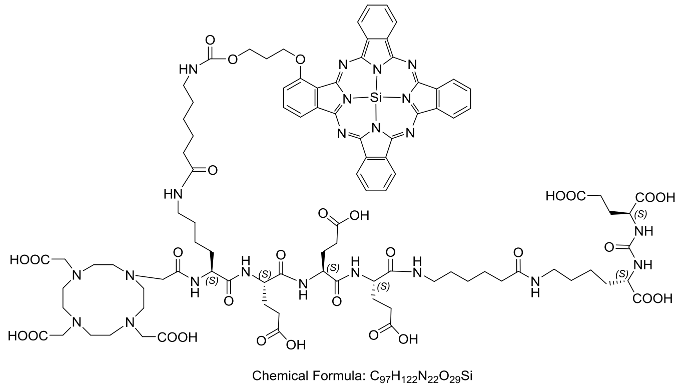

Chemical Formula: $C_{97}H_{122}N_{22}O_{29}Si$

m/z: 2087.86 (100.0%), 2086.85 (95.3%), 2088.86 (40.8%), 2089.86 (17.2%)

PSMA-N142

HPLC chromatogram

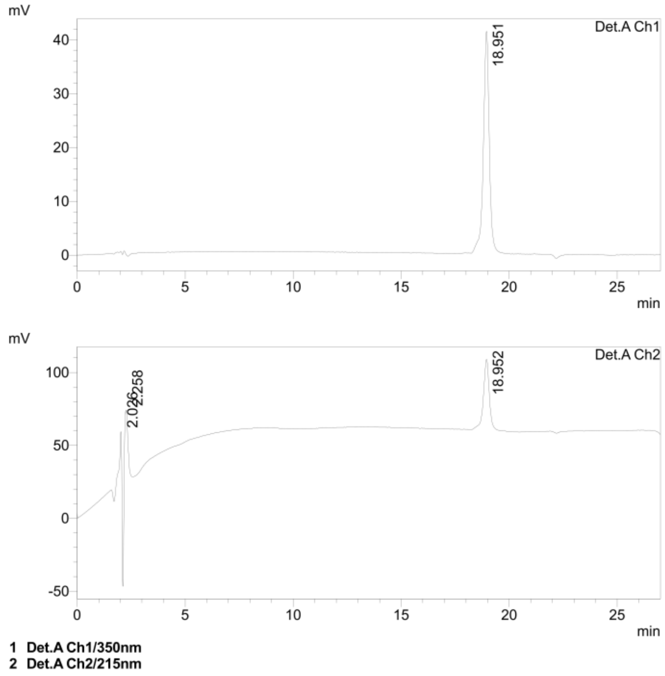

MALDI-ToF spectrum with matrix **HCCA**

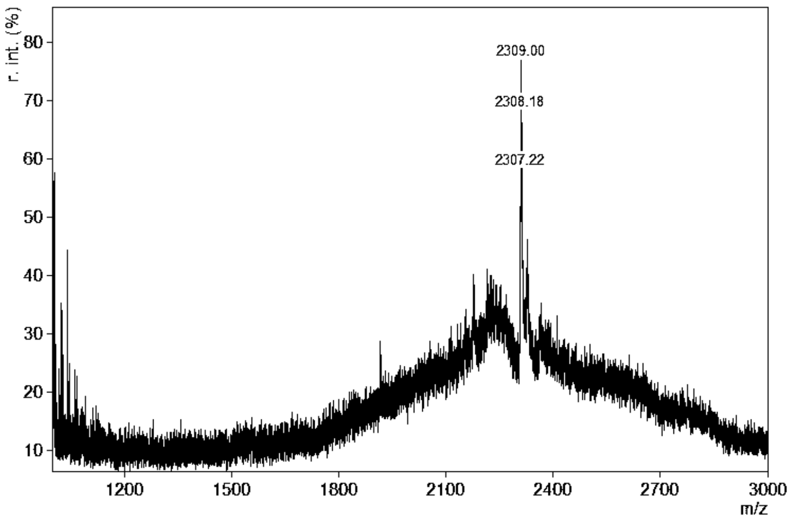

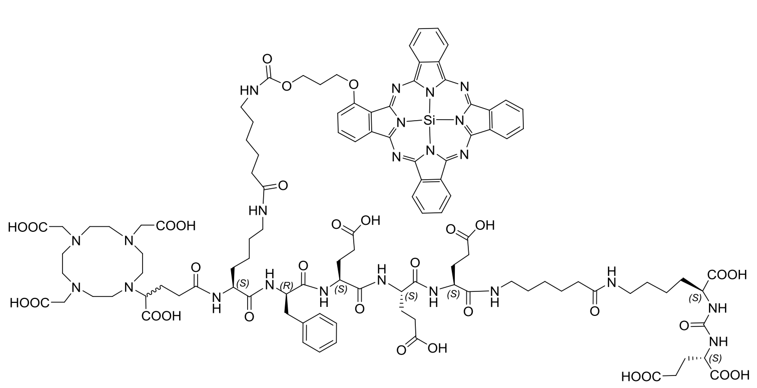

Chemical Formula: $C_{109}H_{135}N_{23}O_{32}Si$

m/z: 2306.94 (100.0%), 2305.94 (84.8%), 2307.95 (58.4%), 2308.95 (12.6%)

PSMA-N143

HPLC chromatogram

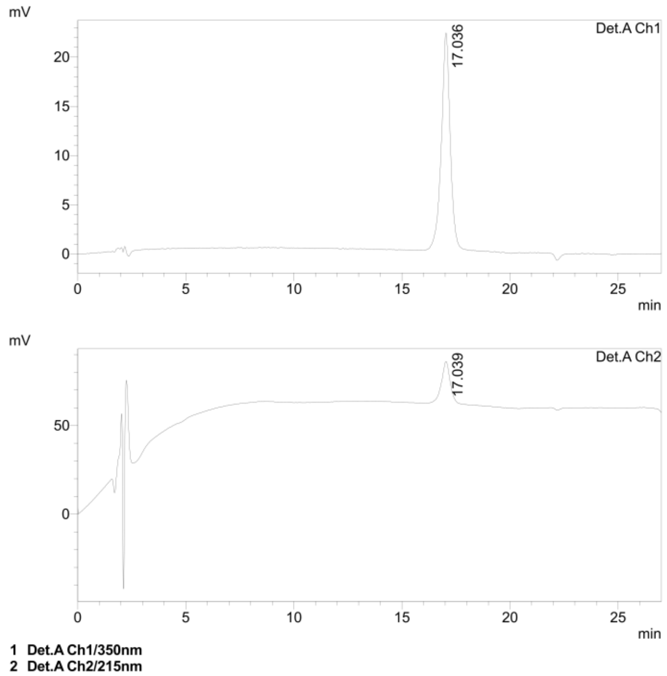

MALDI-ToF spectrum with matrix **HCCA**

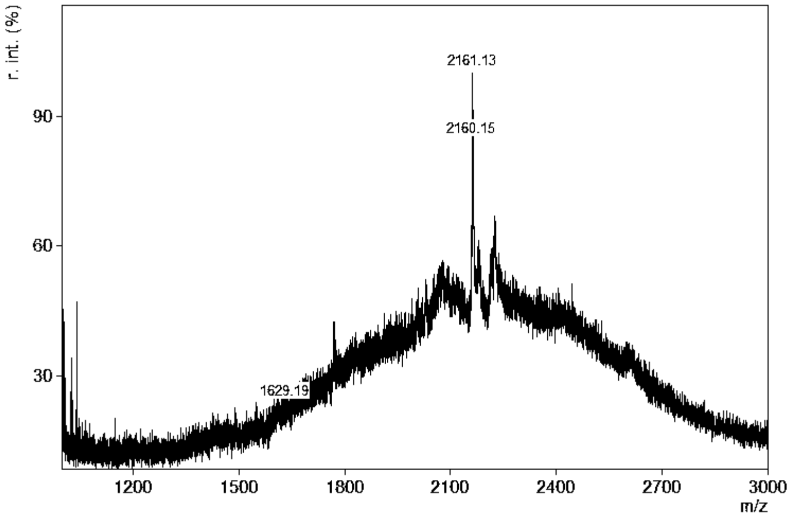

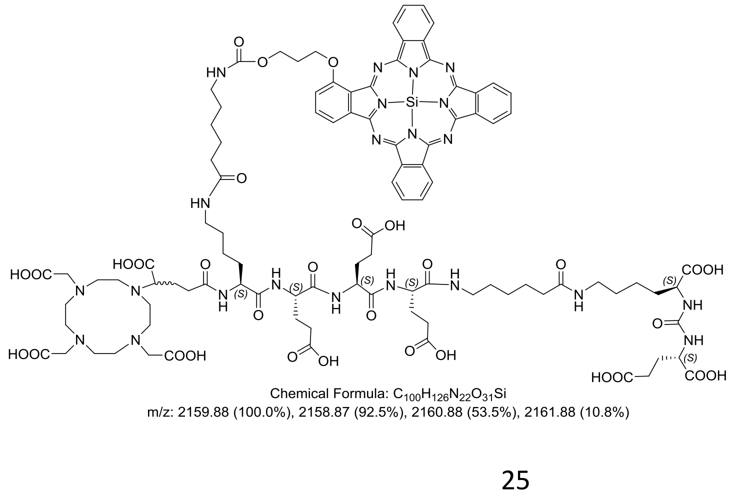

Chemical Formula: $C_{100}H_{126}N_{22}O_{31}Si$
m/z: 2159.88 (100.0%), 2158.87 (92.5%), 2160.88 (53.5%), 2161.88 (10.8%)

PSMA-N144

HPLC chromatogram

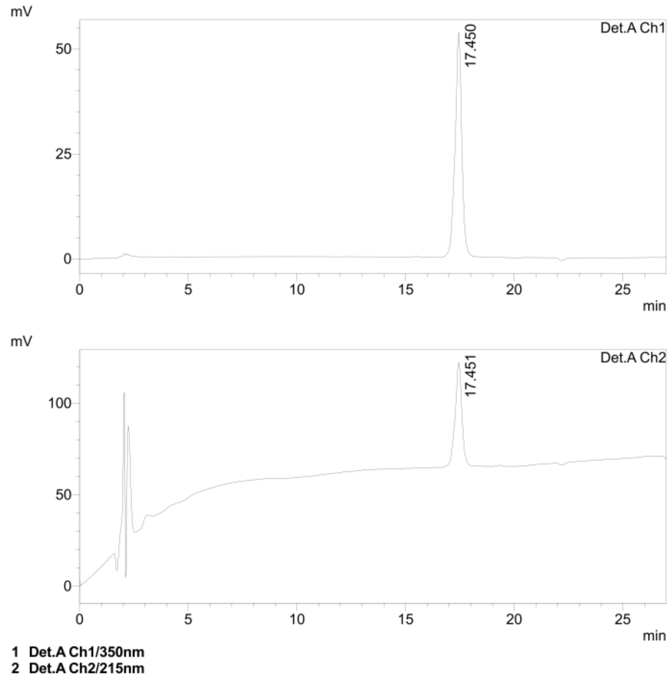

MALDI-ToF spectrum with matrix **HCCA**

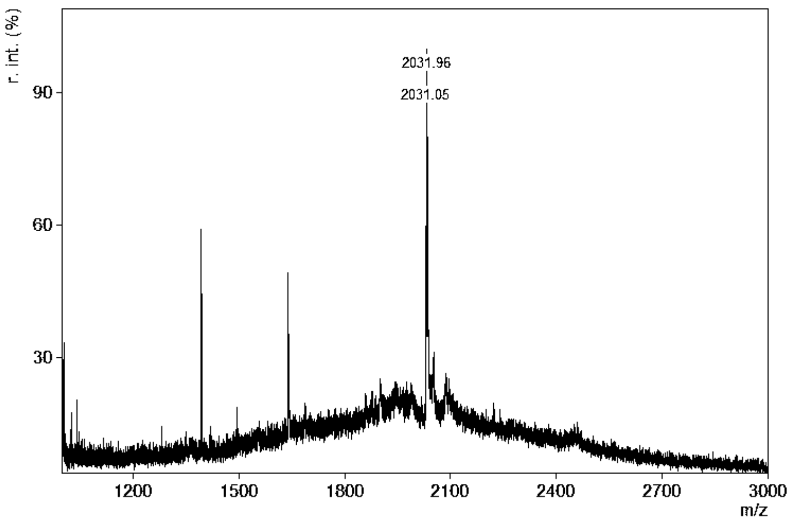

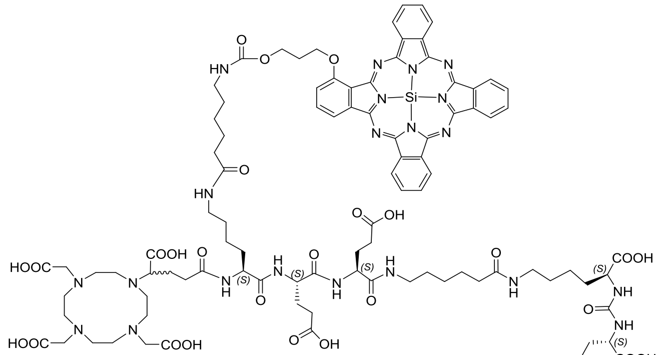

Chemical Formula: $C_{95}H_{119}N_{21}O_{28}Si$

m/z: 2030.83 (100.0%), 2029.83 (97.3%), 2031.84 (41.6%), 2032.84 (11.4%)

References

1. E. Kaiser, R. L. Colescott, C. D. Bossinger, P. I. Cook, Color test for detection of free terminal amino groups in the solid-phase synthesis of peptides. *Anal Biochem* **34**, 595-598 (1970).
